# The Role of Macrophages in Hepatocellular Carcinoma and Their Therapeutic Potential

**DOI:** 10.3390/ijms252313167

**Published:** 2024-12-07

**Authors:** Megan E. Bannister, Devnandan A. Chatterjee, Shishir Shetty, Daniel A. Patten

**Affiliations:** 1 Centre for Liver and Gastrointestinal Research, School of Infection, Inflammation and Immunology, University of Birmingham, Birmingham B15 2TT, UK; 2 National Institute for Health Research, Birmingham Biomedical Research Centre at University Hospitals Birmingham NHS Foundation Trust, Birmingham B15 2TH, UK

**Keywords:** hepatocellular carcinoma, macrophages, TAMs, tumour microenvironment

## Abstract

Hepatocellular carcinoma (HCC) represents a significant clinical burden globally and is predicted to continue to increase in incidence for the foreseeable future. The treatment of HCC is complicated by the fact that, in the majority of cases, it develops on a background of advanced chronic inflammatory liver disease. Chronic inflammation can foster an immunosuppressive microenvironment that promotes tumour progression and metastasis. In this setting, macrophages make up a major immune component of the HCC tumour microenvironment, and in this review, we focus on their contribution to HCC development and progression. Tumour-associated macrophages (TAMs) are largely derived from infiltrating monocytes and their potent anti-inflammatory phenotype can be induced by factors that are found within the tumour microenvironment, such as growth factors, cytokines, hypoxia, and extracellular matrix (ECM) proteins. In general, experimental evidence suggest that TAMs can exhibit a variety of functions that aid HCC tumour progression, including the promotion of angiogenesis, resistance to drug therapy, and releasing factors that support tumour cell proliferation and metastasis. Despite their tumour-promoting profile, there is evidence that the underlying plasticity of these cells can be targeted to help reprogramme TAMs to drive tumour-specific immune responses. We discuss the potential for targeting TAMs therapeutically either by altering their phenotype within the HCC microenvironment or by cell therapy approaches by taking advantage of their infiltrative properties from the circulation into tumour tissue.

## 1. Introduction

Primary liver cancer, also known as hepatocellular carcinoma (HCC), is a major health burden globally, and is currently the third leading cause of cancer deaths [1] with the incidence predicted to rise to 1 million people/year by 2025 [2]. HCC often presents at an advanced stage due to the fact that both cirrhosis and HCC development can be asymptomatic during early stages and there are significant limitations with current screening approaches. Curative treatments, including liver transplantation, are only an option in early-stage disease and transplantation specifically is only undertaken in a small minority (~10%) of patients [3]. Surgical resection is also a highly effective treatment option [4]; however, a large proportion (~90%) of HCC cases develop on a background of chronic inflammatory liver disease in the form of cirrhosis [5], such as viral hepatitis, alcohol-related liver disease (ArLD), or metabolic dysfunction-associated steatohepatitis (MASH), thus significantly limiting patient eligibility. For patients with unresectable HCC tumours, locoregional options, such as ablation, transarterial chemoembolisation (TACE), and selective internal radiation therapy (SIRT), can offer some level of disease control via downstaging tumours or bridging to liver transplantation, but levels of recurrence and metastasis remain high [6,7]. In addition, the existing targeted cancer drugs, such as sorafenib and Lenvatinib, only offer a life extension that is measured in months [8,9] and can be associated with a range of debilitating side effects [10]. Unsurprisingly, the overall 5-year survival rates in HCC patients are extremely poor with a relative 5-year survival of 10–18% [1,11], and there is an urgent need for novel approaches to prevent and treat HCC.

In the last few years, immunotherapy has been a major breakthrough and has now been accepted as the standard of care for patients with advanced HCC [12], but a large majority of patients still fail to respond to this type of therapy [13]. Currently, most immunotherapies target T-cell function (e.g., immune checkpoint inhibitors) and there is gathering evidence that treatment failure is driven by the tumour microenvironment (TME) and, more specifically, immunosuppressive tumour-associated immune cells. Anti-inflammatory immune cell subsets are known to directly block the action of effector T cells and promote tumour growth [13] and are thus proving a major obstacle to effective therapies for the treatment of HCC. Overcoming these immunosuppressive populations within the TME represents a major unmet need in cancer medicine today and novel approaches are required to address this. One of the most abundant immunosuppressive populations present in the HCC TME are TAMs. Here, we discuss the role of TAMs in HCC pathology and explore the potential of targeting and utilising macrophages in the therapeutic treatment of HCC tumours.

## 2. TAMs in the Pathology of HCC

### 2.1. Macrophage Phenotype and Plasticity

Macrophages have traditionally been classified into two distinct phenotypes: proinflammatory and classically activated “M1” macrophages and anti-inflammatory and alternatively activated “M2” macrophages [14,15]. Polarisation into these phenotypes is known to be triggered by stimuli from the microenvironment, such as cytokines and growth factors, which induce the transcription of genes that determine the phenotype and function of the macrophage [16]. Unsurprisingly, M1 macrophages are generally induced by proinflammatory stimuli, such as IFN-γ, TNF-α, GM-CSF, and lipopolysaccharide (LPS) and produce immunostimulatory cytokines such as IL-12, IL-1β, IL-6, and TNF-α [16,17]. M1 macrophages further drive inflammatory responses to both intracellular pathogens and tumourigenic cells through the expression of CD86, iNOS, and MHC-II [18,19] and the induction of Th1 recruitment through the secretion of chemokines, such as CXCL9 and CXCL10 [20]. However, if left unregulated to persist over a sustained period, chronic inflammatory responses can ultimately cause tissue damage, such as fibrosis, to the target organ [21,22]. Conversely, M2 macrophages are induced by anti-inflammatory cytokines, such as IL-4, IL-10, and IL-13 [23,24,25]. M2 macrophages can be further categorised into three sub-phenotypes: M2a, M2b, and M2c. M2a macrophages are specifically induced by IL-4 and IL-13; M2b are induced by exposure to immune complexes, Toll-like receptor (TLR) ligands, or IL-1β; M2c are induced by IL-10 [21]. M2 macrophages are known to produce a repertoire of anti-inflammatory cytokines and chemokines, such as IL-10, TGF-β, CCL17, and CCL22 and can induce Th2 and Treg development and recruitment [22,26]. Additionally, the expression of CD163, CD206, IL-10, and PD-L1 [18,27] defines the M2 phenotype which is associated with dampening inflammatory responses, tissue healing, scavenging debris, and angiogenesis [21,28,29]. Unlike M1s, M2s are poor antigen-presenting cells and suppressors of Th1 responses.

The M1/M2 paradigm is increasingly being challenged as, due to the plasticity of macrophages, the classification into distinct “M1” and “M2” phenotypes is a significant oversimplification and only generally applicable to defined in vitro differentiation experimentation. Therefore, this nomenclature is not fully representative of the spectrum of macrophage activation in vivo or their ability to re-polarise upon the introduction of opposing phenotypic stimuli [14,30,31]. To further emphasise the complexity of macrophage phenotype and plasticity, recent studies have identified another distinct subset of macrophages, in both mouse and human fibrotic tissues, whose phenotype is induced by “type 3” inflammation, including factors such as GM-CSF, IL-17A, and TGF-β1. This subset of so-called “scar-associated macrophages” (SAMs) were shown to actively contribute to fibrotic disease pathology, as they were shown to stimulate hepatic stellate cells to produce collagen I-associated fibrosis but also lack the ability to degrade the collagen I-rich scarring [32]. In addition to this, macrophages found within tumour tissues, termed tumour-associated macrophages (TAMs), are known to possess a unique anti-inflammatory and immunosuppressive phenotype and have been shown to reduce immune surveillance and promote tumour growth and progression [21,29,33].

### 2.2. Monocyte Recruitment to HCC Tumours

**Figure 1:**
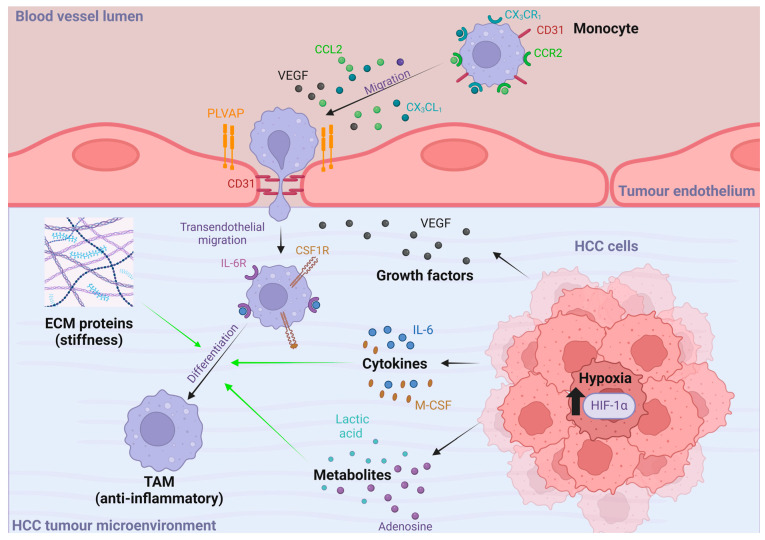
Monocyte recruitment to tumour endothelial cells and the factors that influence their differentiation to TAMs in the HCC tumour microenvironment. Monocytes are recruited to the tumour endothelium via the chemoattractants, CCL2, CX3CL1, and VEGF. Subsequent transendothelial transmigration is mediated by PLVAP and CD31. Once transmigrated into HCC tumour tissues, recruited monocytes encounter a range of stimuli, such as ECM proteins, cytokines, and metabolites, which mediate their differentiation to TAMs. ECM, extracellular matrix; HIF-1α, hypoxia-inducible factor-1α; PLVAP, plasmalemma-vesicle-associated protein; TAM, tumour-associated macrophage; VEGF, vascular endothelial growth factor. Created in BioRender. Kennedy, J. (2024) BioRender.com/l24w527 (accessed 6 November 2024).

A small subset of TAMs in HCC tumours are thought to be derived from the liver-resident macrophage populations, known as Kupffer cells [34]; however, the majority originate from circulating monocytes. Upon entering the tumour microenvironment (TME), the recruited monocytes encounter a wide array of physical (e.g., hypoxia and ECM proteins), chemical (e.g., cytokines (e.g., IL-6), and metabolic (e.g., lactic acid, adenosine, and arginine)) factors known to influence monocyte/macrophage phenotypes [35,36,37]. Ultimately, these tumour-infiltrating monocytes differentiate into the highly anti-inflammatory and immunosuppressive TAMs, which help to sustain the TME and can drive tumour growth and metastasis [38]. Monocytes are recruited to HCC tumours from the systemic circulation via the rich vascular network present in many HCC tumours and cross the tumour endothelial layer, resulting in both peri- and intratumoural populations of TAMs [15,17,31,39,40]. Currently, little is known about the specific mediators of monocyte recruitment to HCC tumours. However, several growth factors, chemokines, and leukocyte adhesion molecules known to play a key role in the recruitment of monocytes to liver tissues during chronic inflammatory disease are expressed in HCC tumours (Figure 1).

In the pathology of chronic liver diseases, hepatocytes, biliary epithelial cells, endothelial cells, monocytes, and macrophages all secrete significant amounts of the angiogenic factor, vascular endothelial growth factor (VEGF) [41,42,43]; in HCC tumours, the tumour cells themselves also release high levels of VEGF [44,45,46,47,48]. In addition to its potent angiogenic properties, VEGF has also been shown to possess chemotactic activity, mediating the migration of monocytes across endothelial cells [49,50]. Indeed, in murine models of skin cancer, VEGF has been shown to play a key role in the accumulation of TAMs [51,52,53], but evidence for this role in HCC tumours is currently lacking. VEGF inhibitors are commonly utilised clinically [54] but the evaluation of their biological effects is largely centred around angiogenesis and tumour cell proliferation. However, their use in conjunction with immune checkpoint inhibition has been shown to skew TAM phenotype towards an “M1-like” phenotype, thus reducing immunosuppression and promoting anti-tumour effects [55].

Circulating bone marrow-derived monocytes express the chemokine receptors, CCR2 and CX_3_CR_1_, which are known to play a key role in their migration towards sites of injury within the body [56,57,58]. CCR2 is the cognate receptor to the CC-chemokine, monocyte chemotactic protein 1 (MCP-1), also known as CCL2, which is widely secreted by hepatocytes, Kupffer cells, hepatic stellate cells, liver sinusoidal endothelial cells and HCC tumour cells [59,60,61,62,63]. The CCL2/CCR2 axis has been widely implicated in the recruitment of monocytes to the liver in a range of murine models of acute [64] and chronic liver injuries [64,65,66,67] and HCC [68]. In addition, the expression of CCL2 and the presence of CCR2^+^ monocytes is indicative of poor prognosis in HCC [69]. Another key mediator of monocyte recruitment is the CX3C chemokine, CX_3_CL_1_, also known as Fractalkine, which regulates the chemotaxis of inflammatory cells, such as monocytes, which are one of the major cell types to express the only known receptor, CX_3_CR_1_ [70]. The chemotactic role of CX_3_CL_1_ is mediated by the cleavage of CX_3_CL_1_ into fragments that act as a monocyte chemoattractant [71] and it has been demonstrated that CX_3_CL_1_, along with the adhesion molecule vascular adhesion protein (VAP)-1 mediates the adhesion, arrest, and transendothelial migration of monocytes across the liver sinusoidal endothelium under physiological flow conditions in vitro [72]. CX_3_CL_1_ is known to be upregulated in chronic liver disease and HCC [71,72,73,74,75]; surprisingly, high CX_3_CL_1_ levels in liver cirrhosis and HCC correlated with better patient prognosis [71,75]. This could potentially be explained by CX_3_CL_1_ influencing the phenotype of infiltrating monocytes [71], or the anti-tumourigenic effect also being driven by other CX_3_CR_1_-expressing cell types [70].

The recruitment and migration of circulating monocytes to the liver during acute injury or chronic disease is also mediated by the expression of adhesion molecules on the cell surface of liver endothelial cells. CD31, also known as PECAM-1, is a member of the immunoglobulin superfamily which is constitutively expressed on both endothelial cells and monocytes. CD31–CD31 interactions are vital for monocyte recruitment, adhesion, and paracellular transmigration through endothelial cell tight junctions [76,77,78]. Recently, CD31 was shown to play a key role in the transmigration of monocytes across primary liver endothelial cells under physiological flow conditions in vitro [61]. Plasmalemma vesicle-associated protein (PLVAP) is an endothelial-specific protein and major component of the diaphragms spanning the openings of fenestrae and caveolae in endothelial cells [79,80,81]. PLVAP plays a key role in development, angiogenesis, and vascular permeability, and has a known role in leukocyte trafficking [61,82,83]. Indeed, PLVAP has previously been implicated in the foetal seeding of monocyte-derived macrophages in the liver during development [82] and, more recently, was shown to play a role in monocyte transmigration across primary liver endothelial cells via the regulation of junctional permeability [61,82]. CD31 and PLVAP are both known to be widely expressed on tumour endothelial cells in HCC tumours tissues [84,85,86] and are likely to play [34,87,88] an important role in monocyte recruitment to the TME. Another molecule likely to play a key role in monocyte recruitment to HCC tumours is the classical adhesion molecule, vascular cell adhesion molecule (VCAM)-1. VCAM-1 has previously been implicated in the adhesion of monocytes to conventional vascular endothelial cells, such as human umbilical vein endothelial cells (HUVEC) [89] and human aortic endothelial cells (HAEC) [90], and is highly present on the vasculature and sinusoids of HCC tumours [91].

### 2.3. TAM Differentiation in HCC Tumours

Once monocytes are recruited to HCC tumours, they encounter a range of stimuli, such as cytokines, hypoxia, metabolites, and extracellular matrix proteins, within the TME which mediate their differentiation from monocytes to the potent anti-inflammatory TAM phenotype. The cytokines IL-6 and macrophage colony stimulating factor (M-CSF) are key to this process and are known to be present in significant numbers in the HCC TME. Both IL-6 and M-CSF can bind to receptors on monocytes/macrophages to promote polarisation towards the immunosuppressive “M2-like” TAM phenotype [92,93]. Nevertheless, it is perhaps the more physical aspects of the TME that have the most pronounced effect on monocyte differentiation and TAM phenotype, and these are discussed below.

*TAMs and hypoxia in HCC*: A key hallmark of the TME in HCC tumours is hypoxia. Chronic inflammation and cirrhosis both cause damage to the liver’s vascular network and disrupt blood flow and oxygen delivery to the tissue [94,95]. Additionally, high HCC cell proliferation depletes the oxygen levels in the surrounding microenvironment and exacerbates local hypoxic conditions [94,96] A key transcription factor regulated in response to hypoxia, hypoxia-inducible factor-1α (HIF-1α), is significantly upregulated in HCC and correlates with poor patient outcome [45,97]. It has previously been shown that chronic hypoxic conditions, such as those found within a large majority of HCC tumours, are able to induce the TAM “M2-like” phenotype, resulting in an upregulation and secretion of IL-1β [98]. Increased IL-1β drives the further upregulation of HIF-1α in HCC cells, mediating an epithelial-to-mesenchymal transition (EMT), leading to more aggressive tumours and metastasis [98]. In addition, hypoxia induces HMGB1 expression in mouse and human HCC cells in a HIF-1α-dependent manner, driving TAM recruitment and polarisation and elevating IL-6 concentrations, which further exacerbates EMT, vascular invasion, and the metastasis of HCC tumours [99]. Hypoxic HCC cells also have altered adenosine metabolism, with the production of adenosine monophosphate (AMP) being inhibited; consequently, adenosine accumulates intracellularly and, eventually, is passively excreted into the TME [100,101]. Adenosine is a potent polarising agent in macrophages and is also known to dampen T lymphocyte proliferation [101], has been implicated in the establishment of the immunosuppressive TME in HCC, and has been shown to reduce the efficacy of immune checkpoint inhibitors [95,100,102].

Hypoxia-induced glycolytic metabolism also causes the accumulation of lactic acid within the microenvironment of solid tumours, such as HCC [103,104]. It has only recently been recognised that lactate possesses potent bioactivity and is now known to be able to metabolically reprogramme monocytes/macrophages to the highly anti-inflammatory “M2-like” TAM phenotype [105,106]. Increased and hypoxia-induced lactate production by HCC cells is highly likely to be a major contributor to the upregulation of VEGF and arginase (ARG1) expression in HCC TAMs, pushing their polarisation towards the highly anti-inflammatory “M2-like” phenotype [45,107]. The importance of lactate signalling in HCC TAMs was recently highlighted by Han et al. who showed that nanoparticles targeted specifically to TAMs and loaded with d-lactate, the gut microbe-derived isomer of lactate, were able to reprogramme the immunosuppressive microenvironment of HCC tumours in mice [95,108].

*TAM metabolism in HCC:* In addition to the cellular responses to the hypoxic microenvironment, the inherent rapid division of cancer cells also actively contributes to the levels of a number of key metabolites within the HCC TME, thus further influencing TAM phenotypes. Rapidly dividing cancer cells alter their metabolism to utilise aerobic glycolysis, over oxidative phosphorylation, as their source of energy to facilitate their high nutrient demand and proliferation [109]. This change in respiration pathway leads to a depletion of extracellular metabolites, such as glucose, ATP, lipids, amino acids, and nucleotides, and the further accumulation of lactate within the TME [109,110]. To circumvent competition for glucose and other nutrients, immune cells are known to undergo metabolic reprogramming and phenotypic changes, with macrophages being no exception to this. Through the activation of the mammalian target of the rapamycin (mTOR) pathway, anti-inflammatory TAMs downregulate their glucose metabolism which further perpetuates their anti-inflammatory phenotype and increases glucose availability for cancer cells to utilise, thus promoting tumour proliferation and progression [15,111]. Additionally, TAMs characteristically express CD36 [112,113,114] which allows for the efficient uptake of lipids from the TME and facilitates fatty acid oxidation. Furthermore, “M2-like” TAMs express nuclear receptors, such as peroxisome proliferator-activated receptors (PPAR) and liver x receptors (LXR) [115,116,117], which also enable increased fatty acid oxidation, further driving polarisation towards the “M2-like” phenotype and sustaining the protumour phenotype of the TME [115,116,118]. As mentioned above, the accumulation of lactate within the HCC TME is likely to drive the anti-inflammatory phenotype of TAMs [107]. Under normal homeostatic conditions, systemic lactate concentrations are stringently maintained around 1–2 mM; however, within the TME of some solid tumours, lactate levels can reach up to 40 mM [119]. High lactate concentrations are known to correlate with adverse outcomes in some cancers [119,120] and its profound effects on TAM metabolism and epigenetics [121] inevitably play a key role in this. Nevertheless, the effects of lactate on HCC TAMs are currently woefully understudied. A number of other key metabolites, such as amino acids, are also known to drive the anti-inflammatory TAM phenotype in HCC and this topic has been extensively reviewed recently by Huang et al. [15]; however, other tumour-derived secretory factors are also known to directly influence TAM metabolism. One key study demonstrated that tumour-derived hyaluronan fragments induced the upregulation of a key glycolytic enzyme, PFKFB3, in tumour-infiltrating monocytes/macrophages [122]. This study noted that, along with the metabolic switch to glycolysis, tumour-derived hyaluronan also induced increased expressions of PD-L1 on the tumour-infiltrating monocytes/macrophages in HCC tumours and peritumoural infiltration with PFKFB3^+^CD68^+^ cells correlated with poorer survival in HCC patients [122].

*TAMs and the extracellular matrix in HCC*: Another major factor known to contribute to TAM differentiation within the TME is the ECM. The ECM is a three-dimensional, non-cellular structure in which cells reside in all tissues and organs. As HCC generally develops on a background of chronic liver disease with associated fibrosis, the synthesis and breakdown of ECM proteins are dysregulated, leading to the excessive deposition of ECM proteins, such as collagens and fibronectin [123]. This creates a highly stiff tumour-prone microenvironment, and it has been shown that patients with increased levels of fibrosis have a significantly higher predisposition to developing hepatocellular carcinoma [124]. Increased tissue stiffness, from collagen deposition, and the activation of transcriptional co-activator with a PDZ-binding motif (TAZ) in pre-tumoural hepatocytes drives tumourigenesis [125]. Additionally, the increased stiffness of the ECM in HCC tumours acts as a physical barrier to the migration and infiltration of anti-tumour immune cell subsets, such as NK cells and CD8^+^ T cells [123]. As a result, the dysregulation of the ECM creates the ideal environment for HCC tumourigenesis and distorts the normal movement and functioning of the immune system to respond to tumour development and growth.

ECM scaffolds derived from tumour tissues have recently been shown to actively polarise macrophages into TAMs. Utilising decellularised human omental ovarian metastatic tissues, a recent study showed that monocytes from healthy donor peripheral blood could be cultured with the ECM scaffolds to stimulate a TAM-like macrophage phenotype with significant shared transcriptomic similarities to the actual TAMs found within omental ovarian metastatic tissues [126]. This demonstrates that the extracellular matrix alone can educate macrophages and alter their phenotype. In addition, the physical constraint of macrophages by ECM components, particularly collagens, has also been shown to modulate monocyte/macrophage phenotype. In vitro models have shown that the spatial confinement of macrophages leads to a significant downregulation of proinflammatory responses, in contrast to unconstrained macrophages demonstrating a more proinflammatory phenotype [127]. This has been confirmed by more recent studies that have shown that macrophages grown on softer substrates, i.e., that are less constrained, are primed towards a proinflammatory phenotype [128,129]. Furthermore, a study in breast cancer tissues demonstrated that the stiffness of the TME correlated with the number of TAM infiltrations, with a greater number in more aggressive tumour subtypes [130]. This changes the paradigm that the ECM is merely an inert bystander that acts as a physical support in the TME and highlights that the ECM could significantly contribute to the generation of TAMs in HCC tumours. Thus, the ECM–TAM relationship could be a key driver of the immunosuppressive microenvironment associated with poor prognosis in HCC [131].

### 2.4. The Role of TAMs in HCC Pathology

TAMs represent a major immune component of the TME and it is widely understood that high levels of TAMs correlate with poor patient prognosis and survival in a range of solid tumour cancers, including HCC [45,132,133,134,135]. In addition, the “M2-like” TAMs express low levels of major histocompatibility complex (MHC) class II, conferring low antigen-presenting capabilities, immune stimulation, and cytotoxic abilities [136,137]. TAMs can also actively suppress cytotoxic T-cell activity by hindering trafficking to the tumour site [138] and inducing the upregulation of T-cell checkpoint molecules [139]. In addition, TAMs can deplete key metabolites required for T-cell activation and proliferation from the TME, such as arginase-1 which depletes arginine [140], and actively secrete potent anti-inflammatory cytokines, such as IL-10 and TGF-beta, which inhibit T-cell activation and cytotoxic activity and potentiate the differentiation of regulatory T cells (T_regs_) [141]. Taken all together, these elements of TAM phenotype play a significant role in the generation and maintenance of the immunosuppressive TME with HCC tumours [142].

Another key protumoural trait of TAMs is their promotion of tumour cell proliferation, survival, and metastasis via paracrine signalling. TAMs are known to secrete a vast array of cytokines, chemokines, and growth factors, such as IL-6, IL-8, VEGF, and TGFβ-1, which all promote HCC cell proliferation, EMT, tumour cell migration, and metastasis [133]. TAMs have also been shown to promote HCC cell migration through the activation of the TLR4/STAT3 signalling pathway [143,144]. TLR4 is a tumour stem cell marker and has been found to be upregulated in HCC cells by the secretome of M2 macrophages [144]. The upregulation of TLR4 in HCC cells activates the STAT3 signalling pathway which promotes tumour progression, aggressiveness, and chemoresistance, thereby conferring poor patient prognosis [145,146,147]. TAMs also facilitate the metastasis of HCC cells by producing enzymes, such as MMPs, serine proteases, and cathepsins, which degrade components of the ECM, facilitating tumour cell migration and metastases [148].

TAMs are also known to play an important role in angiogenesis within the TME, producing key angiogenic growth factors, such as vascular endothelial growth factor (VEGF) and platelet-derived growth factor (PDGF) [149,150], and several matrix metalloproteases (MMPs) involved in neovascularisation [151,152]. CCR2^+^ TAMs, which possess a more angiogenic phenotype, are largely present in the dense stromal margins of HCC tumours and are associated with more vascular areas [153]. Furthermore, in a murine model of HCC on a background of fibrosis, the pharmacological inhibition of TAM recruitment via CCL2 showed a significant reduction in the extent of angiogenesis within tumours and, consequently, tumour progression, as measured by tumour volume [153]. TAMs have been identified as a major source of IL-23 in hepatitis B virus (HBV)-mediated HCC, which subsequently drives angiogenesis [154].

TAMs have also been implicated in limiting the effectiveness of HCC treatments and facilitating the development of resistance to therapies. In mice, the depletion of TAMs has been shown to significantly enhance the efficacy of the multikinase inhibitor, sorafenib, against HCC [155]. Moreover, hepatocyte growth factor (HGF) secretion by TAMs has been shown to mediate the development of resistance to sorafenib in HCC cells [156]. In patients with advanced HCC, TACE is a commonly used treatment to try and downstage tumours and the density of TAMs has been shown to correlate with the efficacy of TACE [157]. TACE often employs oxaliplatin-based chemotherapies, and TAMs drive resistance by triggering autophagy in HCC cells, thus circumventing the apoptosis-inducing cytotoxity activity of oxaliplatin [157]. Immune checkpoint blockade in the treatment of HCC has shown limited efficacy to date; consequently, the role of TAMs in this resistance has been investigated. TAMs were recently shown to confer resistance to a programmed cell-death ligand (PD-L1) blockade in a murine model of HCC, driving a highly immunosuppressive TME via the recruitment of T_regs_ [158].

*TAM/HCC tumour cell crosstalk*: TAMs are known to interact with a range of other cell types within the HCC TME, including the tumour cells themselves [159]. The majority of TAM/tumour cell interactions explored within the literature are largely mediated through the secretion of soluble factors, such as cytokines. For example, TAMs are known to produce IL-6, which promotes the expansion of CD44^+^ HCC cancer stem cells (CSCs) in vitro and xenograft tumour growth in vivo [160]. In this study, the tumour-promoting effects of the TAM-derived IL-6 could be abrogated in these models with the addition of the anti-IL-6-receptor therapeutic antibody, Tocilizumab [160]. TAMs also release a number of other inflammatory cytokines, such as IL-1β, IL-8, and TGF- β1, which have been shown to drive the EMT of HCC cells, contributing to more aggressive tumour phenotypes and metastasis [98].

In addition to secreted cytokines, there is increasing interest in the interactions of TAM-derived extracellular vesicles (EVs) with tumour cells, and evidence suggests that they could also promote tumour growth and cancer “stemness” in HCC. The transfer of miRNAs within TAM-derived EVs has been shown to promote the proliferation and stem cell-like properties of HCC cells in vitro [161]. A recent study also implicated TAM-derived EVs in the immune escape and immunotherapy resistance of HCC tumours [162]. In their study, Wang et al. demonstrated that EVs derived from anti-inflammatory macrophages were able to promote the expression of PD-L1 in HCC cells in vitro, via the MISP/IQGAP1 axis, and further confirmed that they facilitated immune escape in vivo in murine HCC models [162]. Furthermore, the TAM/tumour cell crosstalk via EVs is now known to be bidirectional, with emerging evidence indicating that tumour cell-derived EVs and their associated miRNAs contribute to HCC progression by significantly contributing towards the potent anti-inflammatory TAM phenotype. HCC tumour cell-derived EVs have been shown to be enriched in miRNAs, such as miR-146a-5p and miR-23a-3p, which are able to mediate the polarisation of TAMs towards the anti-inflammatory phenotype and upregulate their expression of PD-L1 [163].

*TAM interactions with other cells within the HCC microenvironment*: In addition to interactions with HCC tumour cells themselves, TAMs are also known to interact with other tumour-resident cell types, such as cancer-associated fibroblasts (CAFs). One study showed in vitro that TAM-derived osteopontin mediated the subsequent secretion of oesteopontin from CAFs which, in turn, promoted HCC cell line migration and invasion [164]. It is highly likely that TAMs also influence other key tumour-resident cell types within the HCC TME, such as tumour endothelial cells (TECs); however, studies into such interactions are currently lacking. More widely studied are the interactions between TAMs and other tumour-infiltrating immune cells (TIICs) (reviewed recently by Sung [159].

TAMs express an array of immunosuppressive molecules and cytokines to dampen the anti-tumour activity of cytotoxic natural killer (NK) cells and T cells and maintain a protumourigenic microenvironment [15,165]. For example, TAMs in HCC are known to express CD48 which directly binds to 2B4 (CD244) on NK cells, resulting in NK cell dysfunction [166]. Furthermore, HCC TAMs can directly regulate the tumouricidal activity of CD8^+^ T cells by their expression of PD-L1 [165]; indeed, TAMs are the major expressor of PD-L1 within the HCC TME [122,167,168] and are spatially located in close proximity to infiltrating PD-1^high^TIM3^+^ CD8^+^ T cells [169]. Additionally, TAMs are key players in the failure of immune checkpoint blockade therapy (i.e., anti-PD-L1) in HCC, as they secrete the chemokine CCL20, a potent chemoattractant of CCR6^+^FoxP3^+^ regulatory T cells (T_regs_) [158]. Nevertheless, although the majority of TAMs within the HCC TME possess a potent anti-inflammatory phenotype, there is a small population of proinflammatory “M1-like” macrophages that play a vital role in promoting NK, Th1, and cytotoxic T-cell anti-tumour responses [15,170]. These “M1-like” TAMs secrete proinflammatory cytokines, such as IL-15 and IL-18, which has been shown to induce NK cell activity and the secretion of IFN-γ to promote inflammatory responses and M1-like macrophage polarisation [170,171].

## 3. Macrophage-Based Therapies for HCC

The pivotal role TAMs play in the pathology of HCC highlights their potential as targets for the development of therapies (Table 1). In addition, the capability of macrophages to infiltrate tumours could then act as potential therapeutic targets and drug delivery systems. Below, we discuss the different approaches utilised to develop a macrophage-based therapy for the potential treatment of HCC.

### 3.1. TAM Reduction/Depletion

A common strategy aiming to reduce or deplete TAM populations within solid tumours is the prevention of initial monocyte recruitment to the TME. Targeting the CCL2/CCR2 axis has previously been shown to inhibit HCC tumour growth and metastasis by reducing monocyte and TAM infiltration and increasing CD8^+^ T-cell cytotoxic activity [68]. Examples of CCL2/CCR2 axis antagonists undergoing early phase clinical trials include trabectedin, a CCL2 antagonist used to treat ovarian cancer [172], and cenicriviroc, a CCR2 (and CCR5) inhibitor currently progressing through clinical trials for MASH and fibrosis [173]. Cenicriviroc has previously demonstrated some efficacy in murine models of fatty liver injury [65]; however, it was recently reported to show no efficacy for treating liver fibrosis in MASH patients [174]. Targeting the CCL2/CCR2 has shown some promise in preclinical murine models of HCC, with the CCR2 antagonists, RDC018 and 747, exhibiting good efficacy in an orthotopic liver tumour model, significantly restricting tumour growth and metastasis [68,69]. However, the translatable relevance of the orthotopic liver tumour model utilised in these studies is limited and so blocking the CCL2/CCR2 axis in additional models that more accurately recapitulate human HCC will likely prove more challenging. This is largely due to the fact that Kupffer cells, the tissue-resident macrophage population within the liver, have been shown to self-renew to replenish TAM numbers in mice [175]. Therefore, a therapeutically beneficial level of TAM reduction may not be reached by targeting monocyte recruitment alone. This could potentially be overcome by combination therapies with those targeting Notch signalling [39], chemotherapy, radiation therapy, or other immunotherapies [176,177]. Indeed, an early phase clinical trial (Phase II) is currently ongoing in which the CCR2 antagonist, BMS-813160, is being used in conjunction with the anti-PD-1 monoclonal antibody (mAb), Nivolumab (NCT04123379) [178].

An alternative strategy to reduce levels of TAMs may be to trigger their apoptosis in situ. Bisphosphates, such as clodronate, can be utilised to deplete TAMs as they are toxic to myeloid cells and induce apoptosis upon engulfment [179,180,181]. In addition to depleting TAMs, bisphosphates have added anti-tumour properties such as the inhibition of cell proliferation, tumour cell adhesion, and invasion [182]; the induction of tumour cell apoptosis [183]; and enhancing immune surveillance [184,185]. Recently, the use of clodronate, combined with doxorubicin, showed some efficacy in a murine model of HCC [186], thus providing some evidence that this therapeutic approach could be viable in the context of HCC. In addition, alkylating agents, such as trabectedin, approved for the treatment of liposarcoma and leiomyosarcoma, and lurbinectedin, approved for small cell lung cancer, could hold some promise in the treatment of HCC. They have been shown to modulate the immunosuppressive TME of other tumours by inducing monocyte and TAM apoptosis, decreasing inflammatory molecule expression, and reducing tumour angiogenesis [187,188,189]. Therefore, the depletion of TAMs, combined with cytotoxic activity against liver cancer cells, suggests the potential for alkylating agents, such as trabectedin, as a treatment for HCC [142]. The in situ depletion of TAMs could prove an effective strategy for reducing the immunosuppressive microenvironment in tumours, if specifically targeted to TAMs. However, current approaches to TAM depletion, such as bisphosphate treatment, are relatively indiscriminate in nature and inherently pose significant risks of off-target effects, the primary risk being the inadvertent depletion of immunoprotective cells, which greatly increases host susceptibility to infections [190].

### 3.2. Reprogramming TAMs

Macrophages, including TAMs, are highly plastic and responsive to stimuli within their local microenvironment. Leveraging this plasticity to repolarise TAMs within HCC tumours, from their immunosuppressive and protumourgenic phenotype towards a more proinflammatory and tumouricidal phenotype, may present a realistic therapeutic approach [191]. For example, the highly specific CSF-1R tyrosine kinase inhibitor, PLX3397, has been shown to repolarise TAMs to a more proinflammatory phenotype [192], as well as inhibit macrophage proliferation and increase cytotoxic T-cell tumour infiltration in HCC tumours in mice [193]. In addition, the repolarisation of TAMs in HCC tumours may also be achieved through the targeting of known phenotypic markers. Mannose receptor (MR) is considered to be a definitive marker of TAMs in general [194] and is known to be abundantly expressed on TAMs within HCC tumour tissues; indeed, the presence of MR^+^ TAMs is highly indicative of poor patient prognosis [195,196]. The MR-targeted reprogramming of TAMs, using the synthetic 10-mer RP-182, has shown efficacy in several murine cancer models [197], thus demonstrating the potential for targeting TAM-expressed MR in the context of HCC and the possibilities for future studies.

Another scavenger receptor that has been linked to TAM expression is stabilin-1 [198]. TAM-expressed stabilin-1 positively correlates with immune checkpoint therapy resistance and T-cell dysfunction in various cancers [199] and, mechanistically, has been shown to promote tumour progression in an in vivo model of breast cancer by scavenging anti-tumour factor, and secreted protein, acidic and rich in cysteine (SPARC) [198]. Murine studies have demonstrated a genetic deficiency of stabilin-1 leads to reduced intratumoural “M2” macrophages and FoxP3^+^ T_regs_, highlighting the role of TAM-expressed stabilin-1 holds in promoting the immunosuppressive TME [200]. Furthermore, macrophage-specific genetic deletion stabilin-1 gene reduces tumour growth and metastatic spread [200]. A recent Phase I/II first-in-man clinical trial in advanced solid tumour cancer patients, including HCC, targeted stabilin-1 with a humanised function blocking antibody (bexmarilimab) and demonstrated a significant switch in phenotypes of circulating monocytes and intratumoural macrophages in treatment responders, when compared with non-responders [201]. Promisingly, early results indicate that HCC tumours are particularly responsive to bexmarilimab, with 4 out of 11 (36%) HCC patients demonstrating disease control and exhibiting reduced intratumoural anti-inflammatory and increased proinflammatory/adaptive immune cell subsets [201,202].

Although TAM repolarisation therapies have shown some promise in a range of preclinical settings, their translation to clinical use against HCC may be slightly more complex. This is due largely to the ability of proinflammatory monocyte/macrophages to aid HCC tumour evasion of the host immunity [167]. The repolarisation of anti-inflammatory TAMs to a proinflammatory phenotype will likely induce their intrinsic expression of PD-L1 and potentially induce PD-L1 expression on HCC cells [203]. PD-L1 is a member of the B7 co-signalling molecule family with roles in naïve T-cell stimulation and effector T-cell inhibition [204,205]. PD-L1^+^ monocytes/macrophages present in the stroma of HCC tumours have been shown to bind PD-1^+^ tumour infiltrating cytotoxic T cells and impair their proliferation, activation, and expression of proinflammatory cytokines (e.g., IL-2, IFN-γ), thus suppressing cytotoxic responses and promoting tumour evasion and progression [167]. Consequently, the expression of PD-L1 on TAMs is strongly correlated to increased disease progression and the reduced survival of HCC patients [167]. Therefore, it is likely that TAM repolarisation therapies would need to be used in combination with PD-L1/PD-1 checkpoint inhibitors to increase the efficacy of treatment [203].

**Table 1:** Summary of preclinical studies and clinical trials targeting TAMs in HCC.

Approach	Target	Therapeutic Agent	Outcome	Reference(s)
*TAM reduction/depletion*				
	CCL2/CCR2 axis	747 (+sorafenib)	Preclinical study in mice. 747 exhibited good efficacy of tumour growth suppression in an orthotopic liver tumour model. Effect was mediated by significant reduction in TAMs and expansion of CD8^+^ T cells. 747 also potentiated the anti-tumour effects of sorafenib in the same model.	[68]
		RDC018	Preclinical study in mice. RDC018 effectively inhibited the recruitment of tumour-infiltrating monocytes and the “M2-like” polarisation of TAMs in an orthotopic liver tumour model. This resulted in reversal of the immunosuppression status of the TME and activation of an anti-tumour CD8^+^ T-cell response.	[69]
		BMS-813160 (+anti-PD-1 monoclonal antibody (mAb), Nivolumab)	Phase II clinical trial (currently recruiting).	[176]
	TAM apoptosis	Clodronate (+doxorubicin)	Preclinical study in rats. Clodronate was able to significantly inhibit tumour growth in diethylnitroamine (DEN) model of HCC. Clodronate and doxorubicin had a synergistic effect on tumour growth through a combination of macrophage depletion and tumour cell apoptosis.	[184]
*Reprogramming TAMs*				
	CSF-1R	PLX3397	Preclinical study in mice. PLX3397 skewed TAM phenotype towards “M1-like” phenotype and increased tumour infiltration of cytotoxic CD8^+^ T cells, effectively reducing tumour burden in an orthotopic liver tumour model.	[191]
	Stabilin-1	Bexmarilimab	Phase I/II clinical trial. HCC tumours amongst the most responsive to bexmarilimab, with 4 out of 11 (36%) of HCC patients demonstrating disease control and exhibiting reduced intratumoural anti-inflammatory immune cell subsets and increased proinflammatory/adaptive immune cells.	[199,200]

### 3.3. Macrophage-Based Cell Therapies

To our knowledge, macrophage-based cell therapies have not yet been employed for the treatment of HCC and, currently, macrophage-based cell therapy clinical trials have primarily focused on the regenerative treatment of tissue-destructive diseases, such as osteonecrosis (NCT00505219), critical limb ischemia (NCT01483898), cardiomyopathy (NCT01670981, NCT01020968, and NCT00765518), and stroke (NCT01845350) [206]. These studies all utilised ex vivo polarisation and the subsequent adoptive transfer of macrophages and consisted of Phase II or Phase III clinical trials. The majority of these trials reported positive outcomes, and some also noted a decrease in adverse events in the treatment groups, when compared with the control groups [207,208,209]. A more directly liver-related study undertaken recently has been the MATCH (ISRCTN10368050) Phase I/II clinical trial that utilised autologous monocyte-derived macrophages in patients with compensated liver cirrhosis. The Phase I trial demonstrated the safety of this approach with patients receiving a macrophage infusion remaining transplant-free over the 12-month period of the trial and no adverse effects were recorded [210]. The results from the Phase II randomised controlled trial [211] were recently reported but did not show any significant differences in the primary or secondary outcomes (i.e., a reduction in liver fibrosis) between the macrophage infusion and control groups. However, 5 out of 24 patients in the control group developed severe liver-related complications, whereas none occurred in the 26 patients of the treatment group [211]. These data emphasise the safety of this therapeutic approach in patients with compensated liver cirrhosis. Furthermore, a follow-up Phase I/II study, the EMERALD study (NCT03847428), is due to start recruiting in 2024 which aims to investigate the safety and efficacy of autologous monocyte-derived macrophage infusion in patients with decompensated liver cirrhosis following hospitalisation due to their first decompensation event. Therefore, the use of autologous patient macrophages may present a viable approach in the treatment of HCC. Nevertheless, the isolation of monocytes from HCC patients and the subsequent macrophage replicative potential may hinder scalability, which will potentially limit therapy development and production [212].

Another emerging and exciting macrophage-based cell therapy for the treatment of solid tumours, such as HCC, is the utilisation of chimeric antigen receptor (CAR)-macrophages [213]. This approach uses genetic engineering to express receptors against tumour-expressed antigens, thus specifically targeting macrophages against tumour cells [214,215]. The use of CAR technology in T cells (CAR-T cells) has been successfully employed to treat a number of blood cancers, in particular B-cell lymphoma and B-cell acute lymphoblastic leukaemia [216]. However, the success of CAR T-cell therapies for the treatment of solid tumours remains severely limited, mainly owing to the complexities of the TME and the difficulty in delivering the CAR T cells to the tumour itself [217]. Given the increased propensity for macrophages to be able to readily transmigrate across endothelial barriers and traffic through the matrix-rich and anti-inflammatory TME, it is thought that CAR-macrophages may present a more viable approach to treating solid tumours, such as HCC [213,214]. Indeed, two such studies utilising CAR-macrophages have reached early phase clinical trials (Phase I) [206]. One ongoing clinical trial aims to target human epidermal growth factor receptor (HER)2 solid tumours (NCT04660929), whilst another (now terminated) aimed to target mesothelin in advanced ovarian cancer and peritoneal mesothelioma (NCT03608618). However, to our knowledge, no data have currently been reported from either CAR-macrophage trial. Whilst CAR-macrophages may present a promising therapeutic approach for the treatment of HCC tumours, the efficacy may be restricted by the limited expansion of CAR-macrophages both in vitro during production and in vivo following administration. One potential method to overcome this limitation is the production of induced pluripotent stem cells (iPSCs)-derived macrophage cells (CAR-iMac), which can be rapidly expanded in vitro and have shown efficacy both in vitro and in vivo in mice [186]. As with CAR-T-cell therapies [216], the use of CAR-macrophages comes with significant potential risk of off-target toxicity and adverse effects. CAR-targeted tumour antigens are often also expressed on small subsets of healthy cells, thus creating the potential for the unwanted damage of non-tumourous tissues [213,214]. In addition, anti-CAR immune responses may occur [218], significantly contributing to therapy failure and possibly predisposing to adverse events, such as cytokine-release syndrome [219]; although, there is little conclusive evidence to support the latter [218]. The administration of CAR-macrophages has recently shown efficacy in a range of murine models of liver fibrosis [220] and therefore lays the foundation for the preclinical testing of CAR-macrophages in murine models of HCC.

### 3.4. Macrophages for Therapy Delivery

Macrophages are also promising candidates for the development of drug delivery systems, as they are easily loaded with cargo and possess the ability to infiltrate tumours. Due to their inherent phagocytic capabilities, they can engulf drugs and nanoparticle-based drug carriers, such as liposomes [221,222,223], polymers [221,224], and micelles [221,225,226], and subsequently transport them across tumour endothelia to specifically deliver their payload directly to tumours. Therefore, the use of macrophages as a drug delivery mechanism could circumvent the off-target toxicity issues encountered with conventional chemotherapy drug administration. For example, macrophages can be targeted to tumours to release their drug payloads more locally, thus minimising adverse reactions and maximising therapeutic dose [227]. Nevertheless, there are various factors to consider when utilising macrophages as drug delivery systems; (1) nanoparticle uptake; (2) the effects of cargo on macrophage function and phenotype; (3) specifically targeting macrophages to the tumour site; and (4) the targeted release of the drug at tumour site. Current studies present limited and varying conclusions for these aspects.

1. *Nanoparticle uptake*

To maximise drug delivery in cancer-targeting studies, nanoparticle-based drug carriers (nanocarriers), such as liposomes and micelles, are often employed to improve both the passive targeting of tumours and the active targeting of tumour cells [228]. As many chemotherapy agents are highly cytotoxic, a major benefit of pre-loading them inside nanocarriers when using macrophages as a targeted delivery method is a significant reduction in the chances of macrophage dysfunction or death before delivery to the tumour site [223,227]. In addition, it has been shown that the uptake of encapsulated drugs by macrophages is increased when compared with the free drugs alone [223]. Furthermore, the level of nanoparticle uptake can be further enhanced with an increase in surface charge of the nanoparticle (positive or negative), with the greatest uptake seen with positively charged nanoparticles [229]. Initially, nanoparticle size was also thought to play a key role in their uptake by macrophages, with larger nanoparticles showing the greatest rate of uptake [229,230]. However, this was directly contradicted by a study from Chang et al. [231] which demonstrated that smaller nanoparticles were more effectively taken up by macrophages. Nevertheless, a more recent study, which also included a meta-analysis of particle uptake by macrophages, determined that macrophages do not display any preferential uptake of particles of a particular size over others when challenged with a polydisperse emulsion [232]. However, they did note that, regardless of particle size, the total surface area of internalised particles remains relatively constant and suggested that membrane surface availability was the limiting factor of uptake [232].

2. *Effects of cargo on macrophage function and phenotype*

There is an increasing body of evidence that macrophage/TAM-targeted nanoparticles can have profound effects upon macrophage phenotype and function [233]. However, the consequences of preloading macrophages with nanoparticles for the intended delivery of cargo to tumour tissues is less well understood. Despite anti-inflammatory macrophages being considered more phagocytic in nature, the specific loading of macrophages with nanoparticles does not appear to be influenced by the phenotype or polarisation status of the macrophage [234]. An early study by Chang et al. [231] investigated the effects of nanoparticle size on macrophage function, specifically migration. This study determined that larger nanoparticles increased macrophage migration by increasing CSF-1 receptor expression [231]. The increased expression of CSF-1 receptors will increase responsiveness to the cytokine CSF-1, which is released by HCC tumour cells and is known to mediate macrophage differentiation to the immunosuppressive and protumourigenic “M2-like” TAM phenotype [235]. It was also shown that larger internalised nanoparticles increased the expression of integrins on macrophages, which increases cell migration and adhesion [231]. Conversely, a more recent study by Li et al. [223] determined there was no evidence nanoparticle uptake interfered with or increased the dynamic distribution of macrophages or tumour-targeting ability. This finding is similar to Choi et al. [236] in which macrophages loaded with liposomes containing Doxorubicin were able to migrate and infiltrate lung carcinoma xenograft tumours. In addition, Wendler et al. [234] demonstrated that loading macrophages with nanoparticles (POlyhedrin Delivery System; PODS^®^) had no effect on the motility or migration of macrophages in vitro, and loaded macrophages retained the ability to migrate through narrow pores (8 μm) in response to a chemoattractant, an important consideration for a chemotherapeutic drug delivery system in which macrophages are required to infiltrate tumours.

3. *Specifically targeting macrophage to the tumour site*

Specifically targeting macrophages to the site of solid tumours would be challenging to achieve without some form of genetic manipulation or pre-programming of the macrophages prior to administration. CAR-macrophage-based technologies could potentially be used to home macrophages in on tumour-expressed antigens; however, as mentioned previously, this approach may also come with some off-target toxicity issues [213,214,237]. In mice, the effective tumour targeting of macrophages has previously been achieved by the use of so-called “microrobots” [238]. In this study, primary mouse macrophages were loaded with superparamagnetic nanoparticles (MNPs), intravenously injected in tumour-bearing mice and guided to the tumour site by a combination of external electromagnetic field and chemical gradients released by the tumour cells [238]. Nevertheless, in the context of HCC tumours, the difficulties associated with specifically targeting a therapy towards the liver can be somewhat negated by the method of delivery used. During transarterial chemoembolisation (TACE) treatment, the hepatic artery is catheterised, allowing for the specific delivery of chemotherapies direct to the liver tissues and HCC tumour(s) present. Macrophage-based therapies could potentially be administered using a similar delivery method, thus directly administering the therapy via the blood vessels supplying the HCC tumour.

4. *Targeted release of the cargo at the tumour site*

A vital aspect of utilising macrophages as a drug delivery system is the targeted release of the cargo at the tumour site. Nanocarriers, taken up by phagocytic cells such as macrophages, have shown the potential to provide targeted drug delivery. This can occur via two distinct methods: (1) the internalised drug is released from the nanocarrier into the cytoplasm of the macrophage and then released into the TME by exocytosis or cell lysis from the macrophage, or (2) the nanocarrier itself is expelled from the macrophage at the tumour target site and the drug is directly released into the TME [223]. It has previously been shown that nanocarrier-entrapped drugs release in a more controlled manner and are more likely to be delivered intact. Therefore, drugs contained within nanocarriers are more likely to reach the tumour site when compared with free drugs [223]. Cargo release from some nanocarriers has been shown to be sustained over several days [234] and even weeks [239], thus allowing for more sustained and long-lasting therapeutic effects, rather than a more acute and transient “hit” often associated with some therapies.

## 4. Conclusions

**Figure 2:**
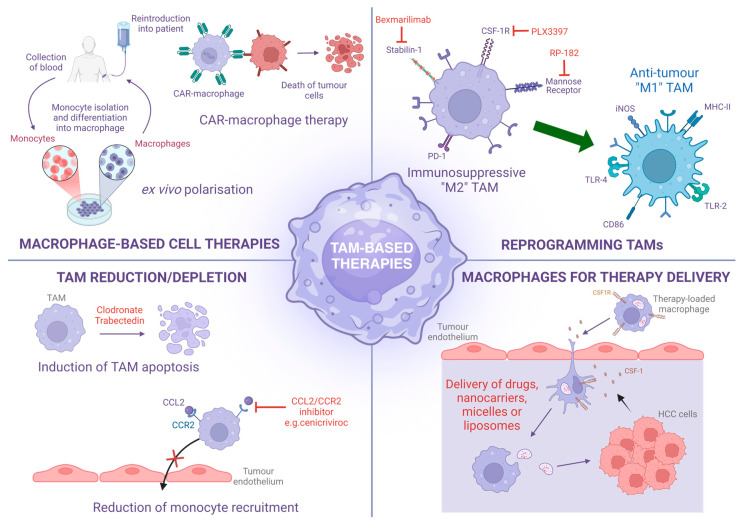
Summary of TAM-based therapies. ***Top left*** Macrophage-based cell therapies include the isolation, the ex vivo polarisation and expansion of autologous macrophages, and the engineering of tumour-targeted CAR-macrophages. ***Top right*** TAMs can be phenotypically reprogrammed by the inhibition/blockade of surface receptors, such as stabilin-1, CSF-1R, and mannose receptor. ***Bottom left*** TAM populations can be reduced by the induction of apoptosis in situ by reagents such as clodronate or trabectedin or depleted by the prevention of monocyte recruitment to the tumour by blockade of the CCL2/CCR2 axis. ***Bottom right*** Macrophages can be utilised as a drug delivery system to specifically target tumours. Macrophages can be preloaded with therapeutic agents and directed to migrate across tumour endothelium and into the tumour tissues, where they release their cargo, e.g., drugs, nanocarriers, micelles, or liposomes. CAR, chimeric antigen receptor; CSF-1, Colony-stimulating factor 1; CSF-1R, Colony-stimulating factor 1 receptor (CSF-1R); TLR, Toll-like receptor. Created in BioRender. Kennedy, J. (2024) BioRender.com/l82y775 (accessed 6 November 2024).

The treatment of HCC is complicated by the fact that a large majority of tumours form on the background of chronic inflammatory liver disease and patients generally present clinically at a late stage. Tumour-infiltrating macrophages (TAMs) are highly abundant in HCC tumours and are known to be key players in the highly complex and immunosuppressive microenvironment that fosters tumour maintenance and growth and, finally, metastatic spread to other sites in the body. Numerous studies have proven that TAMs are instrumental to HCC pathology; therefore, therapeutically targeting them is an attractive prospect (Figure 2). TAMs are largely derived from infiltrating monocytes and one approach to targeting TAMs in HCC is to prevent the migration of monocytes across the tumour endothelial layer. The exact molecular mechanisms involved in this process are yet to be elucidated; however, blocking the CCL2/CCR2 axis has shown some limited efficacy in preclinical studies. Another viable therapeutic approach would be to exploit the highly plastic nature of macrophages to reprogramme TAMs from the immunosuppressive “M2-like” phenotype to a more proinflammatory and anti-tumour phenotype. This approach has recently shown some success in late-stage HCC patients in early phase (Phase I/II) clinical trials, with the scavenger receptor, stabilin-1, targeted by the monoclonal antibody therapy, bexmarilimab. With the recent development of more advanced ‘omics’ technologies such as single-cell RNA sequencing and spatial transcriptomics, which allow the in-depth characterisation of monocyte/macrophage populations in HCC tumours, it is hoped that more TAM-specific targets for the treatment of HCC can be identified. Macrophage-based cellular therapies, such as autologous macrophage infusion or CAR-macrophages, also present a realistic therapeutic approach to the treatment of HCC. Nevertheless, these approaches are extremely expensive and are not without risk of off-target toxicity issues. Finally, utilising macrophages as a therapy delivery system has shown some promise in a range of preclinical studies but has yet to be considered in the treatment of HCC. This method would effectively circumvent the off-target toxicity issues commonly encountered with more conventional treatments (e.g., chemotherapies); however, there are several logistical issues, such as cargo loading/unloading, to consider if this approach is to be utilised.

Overall, it is evident that novel therapeutics for the treatment of HCC are required imminently, with HCC incidence set to rise globally over the next few years and patients currently exhibiting relatively poor prognosis. However, in addition to the development of potential macrophage-based therapies for the treatment of HCC, it is important to consider how these therapies can subsequently be utilised to improve HCC treatment outcomes. This is especially pertinent as most patients fail to respond to current immunotherapy and it is likely macrophage-based therapies will be combined with other therapeutic strategies to boost their synergistic effect. Therefore, whilst we envisage that macrophage/TAM-based therapies will be at the forefront of the emergence of the next wave of immunotherapy technologies for the treatment of HCC, they are likely be utilised in conjunction with existing treatments, such as immune checkpoint blockade and anti-VEGF therapies, to significantly boost the efficacy of these approaches. More specifically, macrophage therapies may be used as a central part of an arsenal, used in combination, priming the TME to boost currently established and future therapeutic targets’ effectiveness and efficiency, overall improving HCC patient outcomes. This has been observed in other solid organ tumours, such as ovarian cancer [240], and is likely to be translated to HCC treatment strategies [240].

## References

[1] Vogel A., Meyer T., Sapisochin G., Salem R., Saborowski A. (2022). Hepatocellular carcinoma. The Lancet.

[2] Office for National Statistics. https://www.ons.gov.uk/.

[3] Llovet J.M., Kelley R.K., Villanueva A., Singal A.G., Pikarsky E., Roayaie S., Lencioni R., Koike K., Zucman-Rossi J., Finn R.S. (2021). Hepatocellular carcinoma. Nat. Rev. Dis. Primers.

[4] Ramesh H. (2014). Resection for hepatocellular carcinoma. J. Clin. Exp. Hepatol..

[5] O’Rourke J.M., Sagar V.M., Shah T., Shetty S. (2018). Carcinogenesis on the background of liver fibrosis: Implications for the management of hepatocellular cancer. World J. Gastroenterol..

[6] Leowattana W., Leowattana T., Leowattana P. (2023). Systemic treatment for unresectable hepatocellular carcinoma. World J. Gastroenterol..

[7] Liapi E., Georgiades C.C., Hong K., Geschwind J.H. (2007). Transcatheter Arterial Chemoembolization: Current Technique and Future Promise. Tech. Vasc. Interv. Radiol..

[8] Dipasquale A., Marinello A., Santoro A. (2021). A Comparison of Lenvatinib versus Sorafenib in the First-Line Treatment of Unresectable Hepatocellular Carcinoma: Selection Criteria to Guide Physician’s Choice in a New Therapeutic Scenario. J. Hepatocell. Carcinoma.

[9] Luo J., Gao B., Lin Z., Fan H., Ma W., Yu D., Yang Q., Tian J., Yang X., Li B. (2022). Efficacy and safety of lenvatinib versus sorafenib in first-line treatment of advanced hepatocellular carcinoma: A meta-analysis. Front. Oncol..

[10] Raoul J., Adhoute X., Penaranda G., Perrier H., Castellani P., Oules V., Bourlière M. (2019). Sorafenib: Experience and Better Manage-ment of Side Effects Improve Overall Survival in Hepatocellular Carcinoma Patients: A Real-Life Retrospective Analysis. Liver Cancer.

[11] Singal A.G., Kanwal F., Llovet J.M. (2023). Global trends in hepatocellular carcinoma epidemiology: Implications for screening, prevention and therapy. Nat. Rev. Clin. Oncol..

[12] Johnston M.P., Khakoo S.I. (2019). Immunotherapy for hepatocellular carcinoma: Current and future. World J. Gastroenterol..

[13] Bai R., Chen N., Li L., Du N., Bai L., Lv Z., Tian H., Cui J. (2020). Mechanisms of Cancer Resistance to Immunotherapy. Front. Oncol..

[14] Das A., Sinha M., Datta S., Abas M., Chaffee S., Sen C.K., Roy S. (2015). Monocyte and Macrophage Plasticity in Tissue Repair and Regeneration. Am. J. Pathol..

[15] Huang J., Wu Q., Geller D.A., Yan Y. (2023). Macrophage metabolism, phenotype, function, and therapy in hepatocellular carcinoma (HCC). J. Transl. Med..

[16] Steevels T.A.M., Meyaard L. (2011). Immune inhibitory receptors: Essential regulators of phagocyte function. Eur. J. Immunol..

[17] Mosser D.M., Edwards J.P. (2008). Exploring the full spectrum of macrophage activation. Nat. Rev. Immunol..

[18] Takiguchi H., Yang C.X., Yang C.W.T., Sahin B., Whalen B.A., Milne S., Akata K., Yamasaki K., Yang J.S.W., Cheung C.Y. (2021). Macrophages with reduced expressions of classical M1 and M2 surface markers in human bronchoalveolar lavage fluid exhibit pro-inflammatory gene signatures. Sci. Rep..

[19] Chávez-Galán L., Olleros M.L., Vesin D., Garcia I. (2015). Much More than M1 and M2 Macrophages, There are also CD169+ and TCR+ Macrophages. Front. Immunol..

[20] Murray P.J., Allen J.E., Biswas S.K., Fisher E.A., Gilroy D.W., Goerdt S., Gordon S., Hamilton J.A., Ivashkiv L.B., Lawrence T. (2014). Macrophage Activation and Polarization: Nomenclature and Experimental Guidelines. Immunity.

[21] Mantovani A., Sica A., Sozzani S., Allavena P., Vecchi A., Locati M. (2004). The chemokine system in diverse forms of macrophage activation and polarization. Trends Immunol..

[22] Sica A., Mantovani A. (2012). Macrophage plasticity and polarization: In vivo veritas. J. Clin. Investig..

[23] Stumpo R., Kauer M., Martin S., Kolb H. (2000). Alternative Activation of Macrophage by IL-10. Pathobiology.

[24] Doyle A.G., Herbein G., Montaner L.J., Minty A.J., Caput D., Ferrara P., Gordon S. (1994). Interleukin-13 alters the activation state of murine macrophages in vitro: Comparison with interleukin-4 and interferon-γ. Eur. J. Immunol..

[25] Stein M., Keshav S., Harris N., Gordon S. (1992). Interleukin 4 potently enhances murine macrophage mannose receptor activity: A marker of alternative immunologic macrophage activation. J. Exp. Med..

[26] Arango Duque G., Descoteaux A. (2014). Macrophage cytokines: Involvement in immunity and infectious diseases. Front. Immunol..

[27] Jayasingam S.D., Citartan M., Thang T.H., Mat Zin A.A., Ang K.C., Ch’ng E.S. (2020). Evaluating the Polarization of Tumor-Associated Macrophages Into M1 and M2 Phenotypes in Human Cancer Tissue: Technicalities and Challenges in Routine Clinical Practice. Front. Oncol..

[28] Arora S., Dev K., Agarwal B., Das P., Syed M.A. (2018). Macrophages: Their role, activation and polarization in pulmonary diseases. Immunobiology.

[29] Ostuni R., Kratochvill F., Murray P.J., Natoli G. (2015). Macrophages and cancer: From mechanisms to therapeutic implications. Trends Immunol..

[30] Ruytinx P., Proost P., Van Damme J., Struyf S. (2018). Chemokine-Induced Macrophage Polarization in Inflammatory Conditions. Front. Immunol..

[31] Hao N., Lü M., Fan Y., Cao Y., Zhang Z., Yang S. (2012). Macrophages in tumor microenvironments and the progression of tumors. Clin. Dev. Immunol..

[32] Fabre F., Barron B., Christensen C., Asano A., Bound B., Lech L., Wadsworth W., Chen C., Wang W., Wang W. (2023). Identification of a broadly fibrogenic macrophage subset induced by type 3 inflammation. Sci. Immunol..

[33] Condeelis J., Pollard J.W. (2006). Macrophages: Obligate Partners for Tumor Cell Migration, Invasion, and Metastasis. Cell.

[34] Sharma A., Seow J.J.W., Dutertre C., Pai R., Blériot C., Mishra A., Wong R.M.M., Singh G.S.N., Sudhagar S., Khalilnezhad S. (2020). Onco-fetal Reprogramming of Endothelial Cells Drives Immunosuppressive Macrophages in Hepatocellular Carcinoma. Cell.

[35] Bai R., Li Y., Jian L., Yang Y., Zhao L., Wei M. (2022). The hypoxia-driven crosstalk between tumor and tumor-associated macrophages: Mechanisms and clinical treatment strategies. Mol. Cancer.

[36] Zou Z., Lin H., Li M., Lin B. (2023). Tumor−associated macrophage polarization in the inflammatory tumor microenvironment. Front. Oncol..

[37] Yu K., Yuan W., Wang H., Li Y. (2024). Extracellular matrix stiffness and tumor-associated macrophage polarization: New fields affecting immune exclusion. Cancer Immunol. Immunother..

[38] Lin Y., Xu J., Lan H. (2019). Tumor-associated macrophages in tumor metastasis: Biological roles and clinical therapeutic applications. J. Hematol. Oncol..

[39] Zheng H., Peng X., Yang S., Li X., Huang M., Wei S., Zhang S., He G., Liu J., Fan Q. (2023). Targeting tumor-associated macrophages in hepatocellular carcinoma: Biology, strategy, and immunotherapy. Cell Death Discov..

[40] Pollard J.W. (2004). Tumour-educated macrophages promote tumour progression and metastasis. Nat. Rev. Cancer.

[41] Deli G., Jin C., Mu R., Yang S., Liang Y., Chen D., Makuuchi M. (2005). Immunohistochemical assessment of angiogenesis in hepatocellular carcinoma and surrounding cirrhotic liver tissues. World J. Gastroenterol..

[42] Mukozu T., Nagai H., Matsui D., Kanekawa T., Sumino Y. (2013). Serum VEGF as a Tumor Marker in Patients with HCV-related Liver Cirrhosis and Hepatocellular Carcinoma. Anticancer. Res..

[43] Assy N., Paizi M., Gaitini D., Baruch Y., Spira G. (1999). Clinical implication of VEGF serum levels in cirrhotic patients with or without portal hypertension. World J. Gastroenterol..

[44] Pinto E., Pelizzaro F., Farinati F., Russo F.P. (2023). Angiogenesis and Hepatocellular Carcinoma: From Molecular Mechanisms to Systemic Therapies. Medicina.

[45] Zhang X., Yu C., Zhao S., Wang M., Shang L., Zhou J., Ma Y. (2023). The role of tumor-associated macrophages in hepatocellular carcinoma progression: A narrative review. Cancer Med..

[46] Rőszer T. (2015). Understanding the Mysterious M2 Macrophage Through Activation Markers and Effector Mechanisms. Mediat. Inflamm..

[47] Mulligan J.K., Rosenzweig S.A., Young M.R.I. (2010). Tumor secretion of VEGF induces endothelial cells to suppress T cell functions through the production of PGE2. J. Immunother..

[48] Funyu J., Mochida S., Inao M., Matsui A., Fujiwara K. (2001). VEGF Can Act as Vascular Permeability Factor in the Hepatic Sinusoids through Upregulation of Porosity of Endothelial Cells. Biochem. Biophys. Res. Commun..

[49] Zittermann S.I., Issekutz A.C. (2006). Endothelial growth factors VEGF and bFGF differentially enhance monocyte and neutrophil recruitment to inflammation. J. Leukoc. Biol..

[50] Heil M., Clauss M., Suzuki K., Buschmann I.R., Willuweit A., Fischer S., Schaper W. (2000). Vascular endothelial growth factor (VEGF) stimulates monocyte migration through endothelial monolayers via increased integrin expression. Eur. J. Cell Biol..

[51] Linde N., Lederle W., Depner S., van Rooijen N., Gutschalk C.M., Mueller M.M. (2012). Vascular endothelial growth factor-induced skin carcinogenesis depends on recruitment and alternative activation of macrophages. J. Pathol..

[52] Lin E.Y., Li J., Bricard G., Wang W., Deng Y., Sellers R., Porcelli S.A., Pollard J.W. (2007). Vascular endothelial growth factor restores delayed tumor progression in tumors depleted of macrophages. Mol. Oncol..

[53] Cursiefen C., Chen L., Borges L.P., Jackson D., Cao J., Radziejewski C., D’Amore P.A., Dana M.R., Wiegand S.J., Streilein J.W. (2004). VEGF-A stimulates lymphangiogenesis and hemangiogenesis in inflammatory neovascularization via macrophage recruitment. J. Clin. Investig..

[54] Boige V., Malka D., Bourredjem A., Dromain C., Baey C., Jacques N., Pignon J., Vimond N., Bouvet-Forteau N., De Baere T. (2012). Efficacy, safety, and biomarkers of single-agent bevacizumab therapy in patients with advanced hepatocellular carcinoma. Oncologist.

[55] Shigeta K., Datta M., Hato T., Kitahara S., Chen I.X., Matsui A., Kikuchi H., Mamessier E., Aoki S., Ramjiawan R.R. (2020). Dual Programmed Death Receptor-1 and Vascular Endothelial Growth Factor Receptor-2 Blockade Promotes Vascular Normalization and Enhances Antitumor Immune Responses in Hepatocellular Carcinoma. Hepatology.

[56] Kratofil R.M., Kubes P., Deniset J.F. (2017). Monocyte Conversion During Inflammation and Injury. Arter. Thromb. Vasc. Biol..

[57] Spahn J.H., Kreisel D. (2014). Monocytes in Sterile Inflammation: Recruitment and Functional Consequences. Arch. Immunol. Ther. Exp..

[58] Brempelis K.J., Crispe I.N. (2016). Infiltrating monocytes in liver injury and repair. Clin. Trans. Immunol..

[59] Bonnardel J., T’Jonck W., Gaublomme D., Browaeys R., Scott C.L., Martens L., Vanneste B., De Prijck S., Nedospasov S.A., Kremer A. (2019). Stellate Cells, Hepatocytes, and Endothelial Cells Imprint the Kupffer Cell Identity on Monocytes Colonizing the Liver Macrophage Niche. Immunity.

[60] Papaioannou S., See J., Jeong M., De La Torre C., Ast V., Reiners-Koch P., Sati A., Mogler C., Platten M., Cerwenka A. (2023). Liver sinusoidal endothelial cells orchestrate NK cell recruitment and activation in acute inflammatory liver injury. Cell Rep..

[61] Wilkinson A.L., Hulme S., Kennedy J.I., Mann E.R., Horn P., Shepherd E.L., Yin K., Zaki M.Y.W., Hardisty G., Lu W. (2023). The senescent secretome drives PLVAP expression in cultured human hepatic endothelial cells to promote monocyte transmigration. iScience.

[62] Yin K., Patten D., Gough S., de Barros Gonçalves S., Chan A., Olan I., Cassidy L., Poblocka M., Zhu H., Lun A. (2022). Senescence-induced endothelial phenotypes underpin immune-mediated senescence surveillance. Genes. Dev..

[63] She S., Ren L., Chen P., Wang M., Chen D., Wang Y., Chen H. (2022). Functional Roles of Chemokine Receptor CCR2 and Its Ligands in Liver Disease. Front. Immunol..

[64] Mossanen J.C., Krenkel O., Ergen C., Govaere O., Liepelt A., Puengel T., Heymann F., Kalthoff S., Lefebvre E., Eulberg D. (2016). Chemokine (C-C motif) receptor 2–positive monocytes aggravate the early phase of acetaminophen-induced acute liver injury. Hepatology.

[65] Krenkel O., Puengel T., Govaere O., Abdallah A.T., Mossanen J.C., Kohlhepp M., Liepelt A., Lefebvre E., Luedde T., Hellerbrand C. (2018). Therapeutic inhibition of inflammatory monocyte recruitment reduces steatohepatitis and liver fibrosis. Hepatology.

[66] Ehling J., Bartneck M., Wei X., Gremse F., Fech V., Möckel D., Baeck C., Hittatiya K., Eulberg D., Luedde T. (2014). CCL2-dependent infiltrating macrophages promote angiogenesis in progressive liver fibrosis. Gut.

[67] Baeck C., Wehr A., Karlmark K.R., Heymann F., Vucur M., Gassler N., Huss S., Klussmann S., Eulberg D., Luedde T. (2012). Pharmacological inhibition of the chemokine CCL2 (MCP-1) diminishes liver macrophage infiltration and steatohepatitis in chronic hepatic injury. Gut.

[68] Yao W., Ba Q., Li X., Li H., Zhang S., Yuan Y., Wang F., Duan X., Li J., Zhang W. (2017). A Natural CCR2 Antagonist Relieves Tumor-associated Macrophage-mediated Immunosuppression to Produce a Therapeutic Effect for Liver Cancer. eBioMedicine.

[69] Li X., Yao W., Yuan Y., Chen P., Li B., Li J., Chu R., Song H., Xie D., Jiang X. (2017). Targeting of tumour-infiltrating macrophages via CCL2/CCR2 signalling as a therapeutic strategy against hepatocellular carcinoma. Gut.

[70] Jung S., Aliberti J., Graemmel P., Sunshine M.J., Kreutzberg G.W., Sher A., Littman D.R. (2000). Analysis of fractalkine receptor CX(3)CR1 function by targeted deletion and green fluorescent protein reporter gene insertion. Mol. Cell Biol..

[71] Karlmark K.R., Zimmermann H.W., Roderburg C., Gassler N., Wasmuth H.E., Luedde T., Trautwein C., Tacke F. (2010). The fractalkine receptor CX3CR1 protects against liver fibrosis by controlling differentiation and survival of infiltrating hepatic monocytes. Hepatology.

[72] Aspinall A.I., Curbishley S.M., Lalor P.F., Weston C.J., Blahova M., Liaskou E., Adams R.M., Holt A.P., Adams D.H. (2010). CX(3)CR1 and vascular adhesion protein-1-dependent recruitment of CD16(+) monocytes across human liver sinusoidal endothelium. Hepatology.

[73] Efsen E., Grappone C., DeFranco R.M.S., Milani S., Romanelli R.G., Bonacchi A., Caligiuri A., Failli P., Annunziato F., Pagliai G. (2002). Up-regulated expression of fractalkine and its receptor CX3CR1 during liver injury in humans. J. Hepatol..

[74] Wasmuth H.E., Zaldivar M.M., Berres M., Werth A., Scholten D., Hillebrandt S., Tacke F., Schmitz P., Dahl E., Wiederholt T. (2008). The fractalkine receptor CX3CR1 is involved in liver fibrosis due to chronic hepatitis C infection. J. Hepatol..

[75] Matsubara T., Ono T., Yamanoi A., Tachibana M., Nagasue N. (2007). Fractalkine-CX3CR1 axis regulates tumor cell cycle and deteriorates prognosis after radical resection for hepatocellular carcinoma. J. Surg. Oncol..

[76] Albelda S.M., Muller W.A., Buck C.A., Newman P.J. (1991). Molecular and cellular properties of PECAM-1 (endoCAM/CD31): A novel vascular cell-cell adhesion molecule. J. Cell Biol..

[77] Schenkel A.R., Mamdouh Z., Muller W.A. (2004). Locomotion of monocytes on endothelium is a critical step during extravasation. Nat. Immunol..

[78] Fu T., Sullivan D.P., Gonzalez A.M., Haynes M.E., Dalal P.J., Rutledge N.S., Tierney A.L., Yescas J.A., Weber E.W., Muller W.A. (2023). Mechanotransduction via endothelial adhesion molecule CD31 initiates transmigration and reveals a role for VEGFR2 in diapedesis. Immunity.

[79] Stan R., Kubitza M., Palade G.E. (1999). PV-1 is a component of the fenestral and stomatal diaphragms in fenestrated endothelia. Proc. Natl. Acad. Sci. USA.

[80] Stan R.V., Tse D., Deharvengt S.J., Smits N.C., Xu Y., Luciano M.R., McGarry C.L., Buitendijk M., Nemani K.V., Elgueta R. (2012). The Diaphragms of Fenestrated Endothelia: Gatekeepers of Vascular Permeability and Blood Composition. Dev. Cell.

[81] Herrnberger L., Seitz R., Kuespert S., Bösl M.R., Fuchshofer R., Tamm E.R. (2012). Lack of endothelial diaphragms in fenestrae and caveolae of mutant Plvap-deficient mice. Histochem. Cell Biol..

[82] Rantakari P., Jäppinen N., Lokka E., Mokkala E., Gerke H., Peuhu E., Ivaska J., Elima K., Auvinen K., Salmi M. (2016). Fetal liver endothelium regulates the seeding of tissue-resident macrophages. Nature.

[83] Keuschnigg J., Henttinen T., Auvinen K., Karikoski M., Salmi M., Jalkanen S. (2009). The prototype endothelial marker PAL-E is a leukocyte trafficking molecule. Blood.

[84] Frachon S., Gouysse G., Dumortier J., Couvelard A., Nejjari M., Mion F., Berger F., Paliard P., Boillot O., Scoazec J. (2001). Endothelial cell marker expression in dysplastic lesions of the liver: An immunohistochemical study. J. Hepatol..

[85] Bösmüller H., Pfefferle V., Bittar Z., Scheble V., Horger M., Sipos B., Fend F. (2018). Microvessel density and angiogenesis in primary hepatic malignancies: Differential expression of CD31 and VEGFR-2 in hepatocellular carcinoma and intrahepatic cholangiocarcinoma. Pathol. Res. Pract..

[86] Qian H., Yang L., Zhao W., Chen H., He S. (2018). A comparison of CD105 and CD31 expression in tumor vessels of hepatocellular carcinoma by tissue microarray and flow cytometry. Exp. Ther. Med..

[87] Wang Y., Cheng T., Chen T., Chang K., Chuang V.P., Kao K. (2014). Plasmalemmal Vesicle Associated Protein (PLVAP) as a therapeutic target for treatment of hepatocellular carcinoma. BMC Cancer.

[88] Aizarani N., Saviano A., Sagar, Mailly L., Durand S., Herman J.S., Pessaux P., Baumert T.F., Grün D. (2019). A human liver cell atlas reveals heterogeneity and epithelial progenitors. Nature.

[89] Carlos T., Kovach N., Schwartz B., Rosa M., Newman B., Wayner E., Benjamin C., Osborn L., Lobb R., Harlan J. (1991). Human monocytes bind to two cytokine-induced adhesive ligands on cultured human endothelial cells: Endothelial-leukocyte adhesion molecule-1 and vascular cell adhesion molecule-1. Blood.

[90] Mohan S., Mohan N., Valente A.J., Sprague E.A. (1999). Regulation of low shear flow-induced HAEC VCAM-1 expression and monocyte adhesion. Am. J. Physiol. -Cell Physiol..

[91] Yoong K.F., McNab G., Hübscher S.G., Adams D.H. (1998). Vascular Adhesion Protein-1 and ICAM-1 Support the Adhesion of Tumor-Infiltrating Lymphocytes to Tumor Endothelium in Human Hepatocellular Carcinoma. J. Immunol..

[92] Zhu X.-D., Zhang J.-B., Zhuang P.-Y., Zhu H.-G., Zhang W., Xiong Y.-Q., Wu W.-Z., Wang L., Tang Z.-Y., Sun H.-C. (2008). High Expression of Macrophage Colony-Stimulating Factor in Peritumoral Liver Tissue Is Associated With Poor Survival After Curative Resection of Hepatocellular Carcinoma. JCO.

[93] Zhang W., Liu Y., Yan Z., Yang H., Sun W., Yao Y., Chen Y., Jiang R. (2020). IL-6 promotes PD-L1 expression in monocytes and macrophages by decreasing protein tyrosine phosphatase receptor type O expression in human hepatocellular carcinoma. J. Immunother. Cancer.

[94] Wu X., Xie G., Chen D. (2007). Hypoxia and hepatocellular carcinoma: The therapeutic target for hepatocellular carcinoma. J. Gastroenterol. Hepatol..

[95] Cheu C., Chiu C., Kwan K., Yang Y., Yuen Y., Goh G., Chui C., Shen S., Law L., Li L. (2023). Hypoxia-inducible factor orchestrates adenosine metabolism to promote liver cancer development. Sci. Adv..

[96] Sin S.Q., Mohan C.D., Goh R.M.W., You M., Nayak S.C., Chen L., Sethi G., Rangappa K.S., Wang L. (2023). Hypoxia signaling in hepatocellular carcinoma: Challenges and therapeutic opportunities. Cancer Metastasis Rev..

[97] Luo D., Wang Z., Wu J., Jiang C., Wu J. (2014). The role of hypoxia inducible factor-1 in hepatocellular carcinoma. Biomed. Res. Int..

[98] Zhang J., Zhang Q., Lou Y., Fu Q., Chen Q., Wei T., Yang J., Tang J., Wang J., Chen Y. (2018). Hypoxia-inducible factor-1α/interleukin-1β signaling enhances hepatoma epithelial–mesenchymal transition through macrophages in a hypoxic-inflammatory microenvironment. Hepatology.

[99] Jiang J., Wang G., Wang Y., Huang H., Li W., Qu X. (2018). Hypoxia-induced HMGB1 expression of HCC promotes tumor invasiveness and metastasis via regulating macrophage-derived IL-6. Exp. Cell Res..

[100] Wang J., Wang Y., Chu Y., Li Z., Yu X., Huang Z., Xu J., Zheng L. (2021). Tumor-derived adenosine promotes macrophage proliferation in human hepatocellular carcinoma. J. Hepatol..

[101] Morello S., Pinto A., Blandizzi C., Antonioli L. (2015). Myeloid cells in the tumor microenvironment: Role of adenosine. Oncoimmunology.

[102] Csóka B., Selmeczy Z., Koscsó B., Németh Z.H., Pacher P., Murray P.J., Kepka-Lenhart D., Morris S.M.J., Gause W.C., Leibovich S.J. (2012). Adenosine promotes alternative macrophage activation via A2A and A2B receptors. FASEB J..

[103] De Matteis S., Ragusa A., Marisi G., De Domenico S., Casadei Gardini A., Bonafè M., Giudetti A.M. (2018). Aberrant Metabolism in Hepatocellular Carcinoma Provides Diagnostic and Therapeutic Opportunities. Oxidative Med. Cell. Longev..

[104] Feng J., Li J., Wu L., Yu Q., Ji J., Wu J., Dai W., Guo C. (2020). Emerging roles and the regulation of aerobic glycolysis in hepatocellular carcinoma. J. Exp. Clin. Cancer Res..

[105] Noe J.T., Rendon B.E., Geller A.E., Conroy L.R., Morrissey S.M., Young L.E.A., Bruntz R.C., Kim E.J., Wise-Mitchell A., Barbosa de Souza Rizzo M. (2021). Lactate supports a metabolic-epigenetic link in macrophage polarization. Sci. Adv..

[106] Zhou H., Yan X.-Y., Yu W., Liang X., Du X., Liu Z., Long J., Zhao G., Liu H. (2022). Lactic acid in macrophage polarization: The significant role in inflammation and cancer. Int. Rev. Immunol..

[107] Colegio O.R., Chu N., Szabo A.L., Chu T., Rhebergen A.M., Jairam V., Cyrus N., Brokowski C.E., Eisenbarth S.C., Phillips G.M. (2014). Functional polarization of tumour-associated macrophages by tumour-derived lactic acid. Nature.

[108] Han H., Bao B., Zou Z., Wang W., Li L., Yang Y., Liao L., Zhang Z., Jiang J., Liang L. (2023). d-lactate modulates M2 tumor-associated macrophages and remodels immunosuppressive tumor microenvironment for hepatocellular carcinoma. Sci. Adv..

[109] Vander Heiden V.H., Cantley C., Thompson T. (2009). Understanding the Warburg Effect: The Metabolic Requirements of Cell Proliferation. Science.

[110] Chen P., Zuo H., Xiong H., Kolar M.J., Chu Q., Saghatelian A., Siegwart D.J., Wan Y. (2017). Gpr132 sensing of lactate mediates tumor–macrophage interplay to promote breast cancer metastasis. Proc. Natl. Acad. Sci. USA.

[111] Miller A., Nagy C., Knapp B., Laengle J., Ponweiser E., Groeger M., Starkl P., Bergmann M., Wagner O., Haschemi A. (2017). Exploring Metabolic Configurations of Single Cells within Complex Tissue Microenvironments. Cell Metab..

[112] Su P., Wang Q., Bi E., Ma X., Liu L., Yang M., Qian J., Yi Q. (2020). Enhanced Lipid Accumulation and Metabolism Are Required for the Differentiation and Activation of Tumor-Associated Macrophages. Cancer Res..

[113] Xie Q., Zeng Y., Zhang X., Yu F. (2024). The significance of lipid metabolism reprogramming of tumor-associated macrophages in hepatocellular carcinoma. Cancer Immunol. Immunother..

[114] Yang P., Qin H., Li Y., Xiao A., Zheng E., Zeng H., Su C., Luo X., Lu Q., Liao M. (2022). CD36-mediated metabolic crosstalk between tumor cells and macrophages affects liver metastasis. Nat. Commun..

[115] Ho P., Liu P. (2016). Metabolic communication in tumors: A new layer of immunoregulation for immune evasion. J. Immunother. Cancer.

[116] Sun J., Xu X., Jin L. (2022). Effects of Metabolism on Macrophage Polarization Under Different Disease Backgrounds. Front. Immunol..

[117] Sanjay, Park M., Lee H. (2022). Roles of Fatty Acids in Microglial Polarization: Evidence from In Vitro and In Vivo Studies on Neurodegenerative Diseases. Int. J. Mol. Sci..

[118] Zhang Q., Wang H., Mao C., Sun M., Dominah G., Chen L., Zhuang Z. (2018). Fatty acid oxidation contributes to IL-1β secretion in M2 macrophages and promotes macrophage-mediated tumor cell migration. Mol. Immunol..

[119] Walenta S., Wetterling M., Lehrke M., Schwickert G., Sundfør K., Rofstad E.K., Mueller-Klieser W. (2000). High lactate levels predict likelihood of metastases, tumor recurrence, and restricted patient survival in human cervical cancers. Cancer Res..

[120] Fischer K., Hoffmann P., Voelkl S., Meidenbauer N., Ammer J., Edinger M., Gottfried E., Schwarz S., Rothe G., Hoves S. (2007). Inhibitory effect of tumor cell–derived lactic acid on human T cells. Blood.

[121] Tao H., Zhong X., Zeng A., Song L. (2023). Unveiling the veil of lactate in tumor-associated macrophages: A successful strategy for immunometabolic therapy. Front. Immunol..

[122] Chen D., Ning W., Jiang Z., Peng Z., Zhu L., Zhuang S., Kuang D., Zheng L., Wu Y. (2019). Glycolytic activation of peritumoral monocytes fosters immune privilege via the PFKFB3-PD-L1 axis in human hepatocellular carcinoma. J. Hepatol..

[123] Roy A.M., Iyer R., Chakraborty S. (2023). The extracellular matrix in hepatocellular carcinoma: Mechanisms and therapeutic vulnerability. Cell Rep. Med..

[124] Passi M., Zahler S. (2021). Mechano-Signaling Aspects of Hepatocellular Carcinoma. J. Cancer.

[125] Filliol A., Saito Y., Nair A., Dapito D.H., Yu L., Ravichandra A., Bhattacharjee S., Affo S., Fujiwara N., Su H. (2022). Opposing roles of hepatic stellate cell subpopulations in hepatocarcinogenesis. Nature.

[126] Puttock E.H., Tyler E.J., Manni M., Maniati E., Butterworth C., Burger Ramos M., Peerani E., Hirani P., Gauthier V., Liu Y. (2023). Extracellular matrix educates an immunoregulatory tumor macrophage phenotype found in ovarian cancer metastasis. Nat. Commun..

[127] Jain N., Vogel V. (2018). Spatial confinement downsizes the inflammatory response of macrophages. Nat. Mater..

[128] Chen M., Zhang Y., Zhou P., Liu X., Zhao H., Zhou X., Gu Q., Li B., Zhu X., Shi Q. (2020). Substrate stiffness modulates bone marrow-derived macrophage polarization through NF-κB signaling pathway. Bioact. Mater..

[129] Chuang Y., Chang H., Li C., Cui Y., Lee C., Chen C. (2020). Reactive Oxygen Species and Inflammatory Responses of Macrophages to Substrates with Physiological Stiffness. ACS Appl. Mater. Interfaces.

[130] Acerbi I., Cassereau L., Dean I., Shi Q., Au A., Park C., Chen Y.Y., Liphardt J., Hwang E.S., Weaver V.M. (2015). Human breast cancer invasion and aggression correlates with ECM stiffening and immune cell infiltration. Integr. Biol..

[131] Yeung O.W.H., Lo C., Ling C., Qi X., Geng W., Li C., Ng K.T.P., Forbes S.J., Guan X., Poon R.T.P. (2015). Alternatively activated (M2) macrophages promote tumour growth and invasiveness in hepatocellular carcinoma. J. Hepatol..

[132] Bingle L., Brown N.J., Lewis C.E. (2002). The role of tumour-associated macrophages in tumour progression: Implications for new anticancer therapies. J. Pathol..

[133] Huang Y., Ge W., Zhou J., Gao B., Qian X., Wang W. (2021). The Role of Tumor Associated Macrophages in Hepatocellular Carcinoma. J. Cancer.

[134] Zhou D., Luan J., Huang C., Li J. (2021). Tumor-Associated Macrophages in Hepatocellular Carcinoma: Friend or Foe?. Gut Liver.

[135] Tian Z., Hou X., Liu W., Han Z., Wei L. (2019). Macrophages and hepatocellular carcinoma. Cell Biosci..

[136] Van Ginderachter J.A., Movahedi K., Hassanzadeh Ghassabeh G., Meerschaut S., Beschin A., Raes G., De Baetselier P. (2006). Classical and alternative activation of mononuclear phagocytes: Picking the best of both worlds for tumor promotion. Immunobiology.

[137] Mantovani A., Sozzani S., Locati M., Allavena P., Sica A. (2002). Macrophage polarization: Tumor-associated macrophages as a paradigm for polarized M2 mononuclear phagocytes. Trends Immunol..

[138] Petty A.J., Li A., Wang X., Dai R., Heyman B., Hsu D., Huang X., Yang Y. (2019). Hedgehog signaling promotes tumor-associated macrophage polarization to suppress intratumoral CD8+ T cell recruitment. J. Clin. Investig..

[139] Petty A.J., Owen D.H., Yang Y., Huang X. (2021). Targeting Tumor-Associated Macrophages in Cancer Immunotherapy. Cancers.

[140] Geiger R., Rieckmann J.C., Wolf T., Basso C., Feng Y., Fuhrer T., Kogadeeva M., Picotti P., Meissner F., Mann M. (2016). L-Arginine Modulates T Cell Metabolism and Enhances Survival and Anti-tumor Activity. Cell.

[141] Ouyang W., O’Garra A. (2019). IL-10 Family Cytokines IL-10 and IL-22: From Basic Science to Clinical Translation. Immunity.

[142] Lu C., Rong D., Zhang B., Zheng W., Wang X., Chen Z., Tang W. (2019). Current perspectives on the immunosuppressive tumor microenvironment in hepatocellular carcinoma: Challenges and opportunities. Mol. Cancer.

[143] Papadakos S.P., Arvanitakis K., Stergiou I.E., Lekakis V., Davakis S., Christodoulou M., Germanidis G., Theocharis S. (2023). The Role of TLR4 in the Immunotherapy of Hepatocellular Carcinoma: Can We Teach an Old Dog New Tricks?. Cancers.

[144] Yao R., Li J., Zhang R., Chen R., Wang Y. (2018). M2-polarized tumor-associated macrophages facilitated migration and epithelial-mesenchymal transition of HCC cells via the TLR4/STAT3 signaling pathway. World J. Surg. Oncol..

[145] Liu W., Jing Y., Yu G., Han Z., Yu D., Fan Q., Ye F., Li R., Gao L., Zhao Q. (2015). Toll like receptor 4 facilitates invasion and migration as a cancer stem cell marker in hepatocellular carcinoma. Cancer Lett..

[146] Machida K., Feldman D.E., Tsukamoto H. (2015). TLR4-dependent tumor-initiating stem cell-like cells (TICs) in alcohol-associated hepatocellular carcinogenesis. Adv. Exp. Med. Biol..

[147] Eiró N., Altadill A., Juárez L.M., Rodríguez M., González L.O., Atienza S., Bermúdez S., Fernandez-Garcia B., Fresno-Forcelledo M., Rodrigo L. (2014). Toll-like receptors 3, 4 and 9 in hepatocellular carcinoma: Relationship with clinicopathological characteristics and prognosis. Hepatol. Res..

[148] Pan Y., Yu Y., Wang X., Zhang T. (2020). Tumor-Associated Macrophages in Tumor Immunity. Front. Immunol..

[149] Zhu A.X., Duda D.G., Sahani D.V., Jain R.K. (2011). HCC and angiogenesis: Possible targets and future directions. Nat. Rev. Clin. Oncol..

[150] Morse M.A., Sun W., Kim R., He A.R., Abada P.B., Mynderse M., Finn R.S. (2019). The Role of Angiogenesis in Hepatocellular Carcinoma. Clin. Cancer Res..

[151] Quintero-Fabián S., Arreola R., Becerril-Villanueva E., Torres-Romero J., Arana-Argáez V., Lara-Riegos J., Ramírez-Camacho M.A., Alvarez-Sánchez M.E. (2019). Role of Matrix Metalloproteinases in Angiogenesis and Cancer. Front. Oncol..

[152] Scheau C., Badarau I.A., Costache R., Caruntu C., Mihai G.L., Didilescu A.C., Constantin C., Neagu M. (2019). The Role of Matrix Metalloproteinases in the Epithelial-Mesenchymal Transition of Hepatocellular Carcinoma. Anal. Cell. Pathol..

[153] Bartneck M., Schrammen P.L., Möckel D., Govaere O., Liepelt A., Krenkel O., Ergen C., McCain M.V., Eulberg D., Luedde T. (2019). The CCR2+ Macrophage Subset Promotes Pathogenic Angiogenesis for Tumor Vascularization in Fibrotic Livers. Cell. Mol. Gastroenterol. Hepatol..

[154] Zang M., Li Y., He H., Ding H., Chen K., Du J., Chen T., Wu Z., Liu H., Wang D. (2018). IL-23 production of liver inflammatory macrophages to damaged hepatocytes promotes hepatocellular carcinoma development after chronic hepatitis B virus infection. Biochim. Et. Biophys. Acta (BBA) -Mol. Basis Dis..

[155] Zhang W., Zhu X., Sun H., Xiong Y., Zhuang P., Xu H., Kong L., Wang L., Wu W., Tang Z. (2010). Depletion of Tumor-Associated Macrophages Enhances the Effect of Sorafenib in Metastatic Liver Cancer Models by Antimetastatic and Antiangiogenic Effects. Clin. Cancer Res..

[156] Dong N., Shi X., Wang S., Gao Y., Kuang Z., Xie Q., Li Y., Deng H., Wu Y., Li M. (2019). M2 macrophages mediate sorafenib resistance by secreting HGF in a feed-forward manner in hepatocellular carcinoma. Br. J. Cancer.

[157] Fu X., Song K., Zhou J., Shi Y., Liu W., Shi G., Gao Q., Wang X., Ding Z., Fan J. (2019). Tumor-associated macrophages modulate resistance to oxaliplatin via inducing autophagy in hepatocellular carcinoma. Cancer Cell Int..

[158] Wu Q., Zhou W., Yin S., Zhou Y., Chen T., Qian J., Su R., Hong L., Lu H., Zhang F. (2019). Blocking Triggering Receptor Expressed on Myeloid Cells-1-Positive Tumor-Associated Macrophages Induced by Hypoxia Reverses Immunosuppression and Anti-Programmed Cell Death Ligand 1 Resistance in Liver Cancer. Hepatology.

[159] Sung P.S. (2022). Crosstalk between tumor-associated macrophages and neighboring cells in hepatocellular carcinoma. Clin. Mol. Hepatol..

[160] Wan S., Zhao E., Kryczek I., Vatan L., Sadovskaya A., Ludema G., Simeone D.M., Zou W., Welling T.H. (2014). Tumor-associated macrophages produce interleukin 6 and signal via STAT3 to promote expansion of human hepatocellular carcinoma stem cells. Gastroenterology.

[161] Wang Y., Wang B., Xiao S., Li Y., Chen Q. (2019). miR-125a/b inhibits tumor-associated macrophages mediated in cancer stem cells of hepatocellular carcinoma by targeting CD90. J. Cell Biochem..

[162] Wang X., Ye X., Chen Y., Lin J. (2023). Mechanism of M2 type macrophage-derived extracellular vesicles regulating PD-L1 expression via the MISP/IQGAP1 axis in hepatocellular carcinoma immunotherapy resistance. Int. Immunopharmacol..

[163] Liu J., Fan L., Yu H., Zhang J., He Y., Feng D., Wang F., Li X., Liu Q., Li Y. (2019). Endoplasmic Reticulum Stress Causes Liver Cancer Cells to Release Exosomal miR-23a-3p and Up-regulate Programmed Death Ligand 1 Expression in Macrophages. Hepatology.

[164] Tokuda K., Morine Y., Miyazaki K., Yamada S., Saito Y., Nishi M., Tokunaga T., Ikemoto T., Imura S., Shimada M. (2021). The interaction between cancer associated fibroblasts and tumor associated macrophages via the osteopontin pathway in the tumor microenvironment of hepatocellular carcinoma. Oncotarget.

[165] Petty A.J., Dai R., Lapalombella R., Baiocchi R.A., Benson D.M., Li Z., Huang X., Yang Y. (2021). Hedgehog-induced PD-L1 on tumor-associated macrophages is critical for suppression of tumor-infiltrating CD8+ T cell function. JCI Insight.

[166] Wu Y., Kuang D., Pan W., Wan Y., Lao X., Wang D., Li X., Zheng L. (2013). Monocyte/macrophage-elicited natural killer cell dysfunction in hepatocellular carcinoma is mediated by CD48/2B4 interactions. Hepatology.

[167] Kuang D., Zhao Q., Peng C., Xu J., Zhang J., Wu C., Zheng L. (2009). Activated monocytes in peritumoral stroma of hepatocellular carcinoma foster immune privilege and disease progression through PD-L1. J. Exp. Med..

[168] Wu K., Kryczek I., Chen L., Zou W., Welling T.H. (2009). Kupffer cell suppression of CD8+ T cells in human hepatocellular carcinoma is mediated by B7-H1/programmed death-1 interactions. Cancer Res..

[169] Ma J., Zheng B., Goswami S., Meng L., Zhang D., Cao C., Li T., Zhu F., Ma L., Zhang Z. (2019). PD1(Hi) CD8(+) T cells correlate with exhausted signature and poor clinical outcome in hepatocellular carcinoma. J. Immunother. Cancer.

[170] Sajid M., Liu L., Sun C. (2022). The Dynamic Role of NK Cells in Liver Cancers: Role in HCC and HBV Associated HCC and Its Therapeutic Implications. Front. Immunol..

[171] Heusinkveld M., van der Burg S.H. (2011). Identification and manipulation of tumor associated macrophages in human cancers. J. Transl. Med..

[172] Allavena P., Signorelli M., Chieppa M., Erba E., Bianchi G., Marchesi F., Olimpio C.O., Bonardi C., Garbi A., Lissoni A. (2005). Anti-inflammatory Properties of the Novel Antitumor Agent Yondelis (Trabectedin): Inhibition of Macrophage Differentiation and Cytokine Production. Cancer Res..

[173] Anstee Q.M., Neuschwander-Tetri B.A., Wong V.W., Abdelmalek M.F., Younossi Z.M., Yuan J., Pecoraro M.L., Seyedkazemi S., Fischer L., Bedossa P. (2020). Cenicriviroc for the treatment of liver fibrosis in adults with nonalcoholic steatohepatitis: AURORA Phase 3 study design. Contemp. Clin. Trials.

[174] Anstee Q.M., Neuschwander-Tetri B.A., Wai-Sun Wong V., Abdelmalek M.F., Rodriguez-Araujo G., Landgren H., Park G.S., Bedossa P., Alkhouri N., Tacke F. (2024). Cenicriviroc Lacked Efficacy to Treat Liver Fibrosis in Nonalcoholic Steatohepatitis: AURORA Phase III Randomized Study. Clin. Gastroenterol. Hepatol..

[175] Ye Y., Zhao J., Lu Y., Gao C., Yang Y., Liang S., Lu Y., Wang L., Yue S., Dou K. (2019). NOTCH Signaling via WNT Regulates the Proliferation of Alternative, CCR2-Independent Tumor-Associated Macrophages in Hepatocellular Carcinoma. Cancer Res..

[176] Nywening T.M., Wang-Gillam A., Sanford D.E., Belt B.A., Panni R.Z., Cusworth B.M., Toriola A.T., Nieman R.K., Worley L.A., Yano M. (2016). Targeting tumour-associated macrophages with CCR2 inhibition in combination with FOLFIRINOX in patients with borderline resectable and locally advanced pancreatic cancer: A single-centre, open-label, dose-finding, non-randomised, phase 1b trial. Lancet Oncol..

[177] Fridlender Z.G., Buchlis G., Kapoor V., Cheng G., Sun J., Singhal S., Crisanti M.C., Wang L.S., Heitjan D., Snyder L.A. (2010). CCL2 Blockade Augments Cancer Immunotherapy. Cancer Res..

[178] Venturini N., Marron T., Casanova-Acebes M., Mandeli J., Doroshow D., Lucas N., Wu K., Hapanowicz O., King P., Hamon P. (2022). 629 Neoadjuvant Nivolumab Combined with CCR2/5 Inhibitor or Anti-IL-8 Antibody in Non-Small Cell Lung Cancer and Hepatocellular Carcinoma. J. Immunother. Cancer.

[179] Diel I.J., Solomayer E., Costa S.D., Gollan C., Goerner R., Wallwiener D., Kaufmann M., Bastert G. (1998). Reduction in New Metastases in Breast Cancer with Adjuvant Clodronate Treatment. N. Engl. J. Med..

[180] Mönkkönen J., Taskinen M., Auriola S.O.K., Urtti A. (1994). Growth Inhibition of Macrophage-Like and Other Cell Types by Liposome-Encapsulated, Calcium-Bound, and Free Bisphosphonates In Vitro. J. Drug Target..

[181] Rogers M.J., Chilton K.M., Coxon F.P., Lawry J., Smith M.O., Suri S., Russel R.G. (1996). Bisphosphonates induce apoptosis in mouse macrophage-like cells in vitro by a nitric oxide-independent mechanism. J. Bone Min. Res..

[182] Tsubaki M., Satou T., Itoh T., Imano M., Ogaki M., Yanae M., Nishida S. (2012). Reduction of metastasis, cell invasion, and adhesion in mouse osteosarcoma by YM529/ONO-5920-induced blockade of the Ras/MEK/ERK and Ras/PI3K/Akt pathway. Toxicol. Appl. Pharmacol..

[183] Tamura T., Shomori K., Nakabayashi M., Fujii N., Ryoke K., Ito H. (2011). Zoledronic acid, a third-generation bisphosphonate, inhibits cellular growth and induces apoptosis in oral carcinoma cell lines. Oncol. Rep..

[184] Van Acker H.H., Anguille S., Willemen Y., Smits E.L., Van Tendeloo V.F. (2016). Bisphosphonates for cancer treatment: Mechanisms of action and lessons from clinical trials. Pharmacol. Ther..

[185] Braza M.S., Klein B. (2013). Anti-tumour immunotherapy with Vγ9Vδ2 T lymphocytes: From the bench to the bedside. Br. J. Haematol..

[186] Zhang H., Sheng D., Han Z., Zhang L., Sun G., Yang X., Wang X., Wei L., Lu Y., Hou X. (2022). Doxorubicin-liposome combined with clodronate-liposome inhibits hepatocellular carcinoma through the depletion of macrophages and tumor cells. Int. J. Pharm..

[187] Germano G., Frapolli R., Belgiovine C., Anselmo A., Pesce S., Liguori M., Erba E., Uboldi S., Zucchetti M., Pasqualini F. (2013). Role of Macrophage Targeting in the Antitumor Activity of Trabectedin. Cancer Cell.

[188] Patel S., Petty W.J., Sands J.M. (2021). An overview of lurbinectedin as a new second-line treatment option for small cell lung cancer. Ther. Adv. Med. Oncol..

[189] Céspedes M.V., Guillén M.J., López-Casas P.P., Sarno F., Gallardo A., Álamo P., Cuevas C., Hidalgo M., Galmarini C.M., Allavena P. (2016). Lurbinectedin induces depletion of tumor-associated macrophages, an essential component of its in vivo synergism with gemcitabine, in pancreatic adenocarcinoma mouse models. Dis. Model. Mech..

[190] Li X., Liu R., Su X., Pan Y., Han X., Shao C., Shi Y. (2019). Harnessing tumor-associated macrophages as aids for cancer immunotherapy. Mol. Cancer.

[191] Khan S.U., Khan M.U., Azhar Ud Din M., Khan I.M., Khan M.I., Bungau S., Hassan S.S.u. (2023). Reprogramming tumor-associated macrophages as a unique approach to target tumor immunotherapy. Front. Immunol..

[192] Fujiwara T., Yakoub M.A., Chandler A., Christ A.B., Yang G., Ouerfelli O., Rajasekhar V.K., Yoshida A., Kondo H., Hata T. (2021). CSF1/CSF1R Signaling Inhibitor Pexidartinib (PLX3397) Reprograms Tumor-Associated Macrophages and Stimulates T-cell Infiltration in the Sarcoma Microenvironment. Mol. Cancer Ther..

[193] Ao J., Zhu X., Chai Z., Cai H., Zhang Y., Zhang K., Kong L., Zhang N., Ye B., Ma D. (2017). Colony-Stimulating Factor 1 Receptor Blockade Inhibits Tumor Growth by Altering the Polarization of Tumor-Associated Macrophages in Hepatocellular Carcinoma. Mol. Cancer Ther..

[194] Gordon S., Martinez F.O. (2010). Alternative activation of macrophages: Mechanism and functions. Immunity.

[195] Ren C., Leng R., Fan Y., Pan H., Li B., Wu C., Wu Q., Wang N., Xiong Q., Geng X. (2017). Intratumoral and peritumoral expression of CD68 and CD206 in hepatocellular carcinoma and their prognostic value. Oncol. Rep..

[196] Dong P., Ma L., Liu L., Zhao G., Zhang S., Dong L., Xue R., Chen S. (2016). CD86+/CD206+, Diametrically Polarized Tumor-Associated Macrophages, Predict Hepatocellular Carcinoma Patient Prognosis. Int. J. Mol. Sci..

[197] Jaynes J., Sable S., Ronzetti R., Bautista B., Knotts K., Abisoye-Ogunniyan A., Li L., Calvo C., Dashnyam D., Singh S. (2020). Mannose receptor (CD206) activation in tumor-associated macrophages enhances adaptive and innate antitumor immune responses. Sci. Transl. Med..

[198] Riabov V., Yin S., Song B., Avdic A., Schledzewski K., Ovsiy I., Gratchev A., Llopis Verdiell M., Sticht C., Schmuttermaier C. (2016). Stabilin-1 is expressed in human breast cancer and supports tumor growth in mammary adenocarcinoma mouse model. Oncotarget.

[199] Hollmén M., Figueiredo C.R., Jalkanen S. (2020). New tools to prevent cancer growth and spread: A ‘Clever’ approach. Br. J. Cancer.

[200] Karikoski M., Marttila-Ichihara F., Elima K., Rantakari P., Hollmén M., Kelkka T., Gerke H., Huovinen V., Irjala H., Holmdahl R. (2014). Clever-1/Stabilin-1 Controls Cancer Growth and Metastasis. Clin. Cancer Res..

[201] Rannikko J.H., Verlingue L., De Miguel M., Pasanen A., Robbrecht D., Skytta T., Iivanainen S., Shetty S., Ma Y.T., Graham D.M. (2023). Bexmarilimab-induced macrophage activation leads to treatment benefit in solid tumors: The phase I/II first-in-human MATINS trial. Cell Rep. Med..

[202] Virtakoivu R., Rannikko J.H., Viitala M., Vaura F., Takeda A., Lönnberg T., Koivunen J., Jaakkola P., Pasanen A., Shetty S. (2021). Systemic Blockade of Clever-1 Elicits Lymphocyte Activation Alongside Checkpoint Molecule Downregulation in Patients with Solid Tumors: Results from a Phase I/II Clinical Trial. Clin. Cancer Res..

[203] Zong Z., Zou J., Mao R., Ma C., Li N., Wang J., Wang X., Zhou H., Zhang L., Shi Y. (2019). M1 Macrophages Induce PD-L1 Expression in Hepatocellular Carcinoma Cells Through IL-1β Signaling. Front. Immunol..

[204] Zang X., Allison J.P. (2007). The B7 Family and Cancer Therapy: Costimulation and Coinhibition. Clin. Cancer Res..

[205] Zou W., Chen L. (2008). Inhibitory B7-family molecules in the tumour microenvironment. Nat. Rev. Immunol..

[206] Na Y.R., Kim S.W., Seok S.H. (2023). A new era of macrophage-based cell therapy. Exp. Mol. Med..

[207] Danon D., Madjar J., Edinov E., Knyszynski A., Brill S., Diamantshtein L., Shinar E. (1997). Treatment of human ulcers by application of macrophages prepared from a blood unit. Exp. Gerontol..

[208] Henry T.D., Traverse J.H., Hammon B.L., East C.A., Bruckner B., Remmers A.E., Recker D., Bull D.A., Patel A.N. (2014). Safety and Efficacy of Ixmyelocel-T. Circ. Res..

[209] Chernykh E.R., Shevela E.Y., Starostina N.M., Morozov S.A., Davydova M.N., Menyaeva E.V., Ostanin A.A. (2016). Safety and Therapeutic Potential of M2 Macrophages in Stroke Treatment. Cell Transpl..

[210] Moroni F., Dwyer B.J., Graham C., Pass C., Bailey L., Ritchie L., Mitchell D., Glover A., Laurie A., Doig S. (2019). Safety profile of autologous macrophage therapy for liver cirrhosis. Nat. Med..

[211] Brennan P.N., MacMillan M., Manship T., Moroni F., Glover A., Graham C., Semple S., Morris D.M., Fraser A.R., Pass C. (2021). Study protocol: A multicentre, open-label, parallel-group, phase 2, randomised controlled trial of autologous macrophage therapy for liver cirrhosis (MATCH). BMJ Open.

[212] Mishra A.K., Malonia S.K. (2023). Advancing cellular immunotherapy with macrophages. Life Sci..

[213] Chen Y., Yu Z., Tan X., Jiang H., Xu Z., Fang Y., Han D., Hong W., Wei W., Tu J. (2021). CAR-macrophage: A new immunotherapy candidate against solid tumors. Biomed. Pharmacother..

[214] Hadiloo K., Taremi S., Heidari M., Esmaeilzadeh A. (2023). The CAR macrophage cells, a novel generation of chimeric antigen-based approach against solid tumors. Biomark. Res..

[215] Sloas C., Gill S., Klichinsky M. (2021). Engineered CAR-Macrophages as Adoptive Immunotherapies for Solid Tumors. Front. Immunol..

[216] Cappell K.M., Kochenderfer J.N. (2023). Long-term outcomes following CAR T cell therapy: What we know so far. Nat. Rev. Clin. Oncol..

[217] Maalej K.M., Merhi M., Inchakalody V.P., Mestiri S., Alam M., Maccalli C., Cherif H., Uddin S., Steinhoff M., Marincola F.M. (2023). CAR-cell therapy in the era of solid tumor treatment: Current challenges and emerging therapeutic advances. Mol. Cancer.

[218] Wang S., Yang Y., Ma P., Zha Y., Zhang J., Lei A., Li N. (2022). CAR-macrophage: An extensive immune enhancer to fight cancer. EBioMedicine.

[219] Shimabukuro-Vornhagen A., Gödel P., Subklewe M., Stemmler H.J., Schlößer H.A., Schlaak M., Kochanek M., Böll B., von Bergwelt-Baildon M.S. (2018). Cytokine release syndrome. J. Immunother. Cancer.

[220] Dai H., Zhu C., Huai Q., Xu W., Zhu J., Zhang X., Zhang X., Sun B., Xu H., Zheng M. (2024). Chimeric antigen receptor-modified macrophages ameliorate liver fibrosis in preclinical models. J. Hepatol..

[221] Pei Y., Yeo Y. (2016). Drug delivery to macrophages: Challenges and opportunities. J. Control. Release.

[222] Liang T., Zhang R., Liu X., Ding Q., Wu S., Li C., Lin Y., Ye Y., Zhong Z., Zhou M. (2021). Recent Advances in Macrophage-Mediated Drug Delivery Systems. Int. J. Nanomed..

[223] Li S., Feng S., Ding L., Liu Y., Zhu Q., Qian Z., Gu Y. (2016). Nanomedicine engulfed by macrophages for targeted tumor therapy. Int. J. Nanomed..

[224] Shofolawe-Bakare O.T., de Mel J.U., Mishra S.K., Hossain M., Hamadani C.M., Pride M.C., Dasanayake G.S., Monroe W., Roth E.W., Tanner E.E.L. (2022). ROS-Responsive Glycopolymeric Nanoparticles for Enhanced Drug Delivery to Macrophages. Macromol. Biosci..

[225] Liu H., Lv H., Duan X., Du Y., Tang Y., Xu W. (2023). Advancements in Macrophage-Targeted Drug Delivery for Effective Disease Management. Int. J. Nanomed..

[226] Ghezzi M., Pescina S., Padula C., Santi P., Del Favero E., Cantù L., Nicoli S. (2021). Polymeric micelles in drug delivery: An insight of the techniques for their characterization and assessment in biorelevant conditions. J. Control. Release.

[227] Si J., Shao S., Shen Y., Wang K. (2016). Macrophages as Active Nanocarriers for Targeted Early and Adjuvant Cancer Chemotherapy. Small.

[228] Pérez-Herrero E., Fernández-Medarde A. (2015). Advanced targeted therapies in cancer: Drug nanocarriers, the future of chemotherapy. Eur. J. Pharm. Biopharm..

[229] He C., Hu Y., Yin L., Tang C., Yin C. (2010). Effects of particle size and surface charge on cellular uptake and biodistribution of polymeric nanoparticles. Biomaterials.

[230] Nowacek A.S., Miller R.L., McMillan J., Kanmogne G., Kanmogne M., Mosley R.L., Ma Z., Graham S., Chaubal M., Werling J. (2009). NanoART synthesis, characterization, uptake, release and toxicology for human monocyte-macrophage drug delivery. Nanomedicine.

[231] Chang Y., Guo H., Li J., Song Y., Zhang M., Jin J., Xing G., Zhao Y. (2013). Adjusting the balance between effective loading and vector migration of macrophage vehicles to deliver nanoparticles. PLoS ONE.

[232] Montel L., Pinon L., Fattaccioli J. (2019). A Multiparametric and High-Throughput Assay to Quantify the Influence of Target Size on Phagocytosis. Biophys. J..

[233] Reichel D., Tripathi M., Perez J.M. (2019). Biological Effects of Nanoparticles on Macrophage Polarization in the Tumor Microenvironment. Nanotheranostics.

[234] Wendler W., James J., Jones J., Pernstich P. (2021). Phagocytosed Polyhedrin-Cytokine Cocrystal Nanoparticles Provide Sustained Secretion of Bioactive Cytokines from Macrophages. BioDesign Res..

[235] Wei C., Zhu M., Zhang P., Huang X., Wan J., Yao X., Hu Z., Chai X., Peng R., Yang X. (2022). PKCα/ZFP64/CSF1 axis resets the tumor microenvironment and fuels anti-PD1 resistance in hepatocellular carcinoma. J. Hepatol..

[236] Choi J., Kim H., Ju E.J., Jung J., Park J., Chung H., Lee J.S., Lee J.S., Park H.J., Song S.Y. (2012). Use of macrophages to deliver therapeutic and imaging contrast agents to tumors. Biomaterials.

[237] Abdin S.M., Paasch D., Morgan M., Lachmann N. (2021). CARs and beyond: Tailoring macrophage-based cell therapeutics to combat solid malignancies. J. Immunother. Cancer.

[238] Nguyen V.D., Min H., Kim H.Y., Han J., Choi Y.H., Kim C., Park J., Choi E. (2021). Primary Macrophage-Based Microrobots: An Effective Tumor Therapy In Vivo by Dual-Targeting Function and Near-Infrared-Triggered Drug Release. ACS Nano.

[239] Whitty C., Pernstich C., Marris C., McCaskie A., Jones M., Henson F. (2022). Sustained delivery of the bone morphogenetic proteins BMP-2 and BMP-7 for cartilage repair and regeneration in osteoarthritis. Osteoarthr. Cartil. Open.

[240] Rodriguez-Garcia A., Lynn R.C., Poussin M., Eiva M.A., Shaw L.C., O’Connor R.S., Minutolo N.G., Casado-Medrano V., Lopez G., Matsuyama T. (2021). CAR-T cell-mediated depletion of immunosuppressive tumor-associated macrophages promotes endogenous antitumor immunity and augments adoptive immunotherapy. Nat. Commun..

